# *Burkholderia contaminans* siderophore-rich supernatants suppress post-harvest anthracnose in avocado fruits

**DOI:** 10.1007/s00253-026-13715-2

**Published:** 2026-01-23

**Authors:** Coyolxauhqui Barrera-Galicia, Sergio A. Covarrubias, Juan Armando Flores de la Torre, Héctor A. Peniche-Pávia, John P. Délano-Frier

**Affiliations:** 1 Facultad de Ciencias Químicas, Universidad Autónoma de Zacatecas, Carretera Zacatecas-Guadalajara Km. 6, Ejido “La Escondida”Ciudad Universitaria Campus Siglo XXI, C.P. 98160 Zacatecas, Zacatecas México; 2 Departamento de Biotecnología y Bioquímica, Centro de Investigación y de Estudios Avanzados del IPN, unidad Irapuato, Km 9.6 Libramiento Norte Carretera Irapuato-León, C.P. 36824 Irapuato, Guanajuato México; 3 Departamento de Recursos del Mar, Centro de Investigación y de Estudios Avanzados del IPN, unidad Mérida, Km. 6 Antigua Carretera a Progreso Apdo. Postal 73, Cordemex, 97310 Mérida, Yucatán México

**Keywords:** Anthracnose, *Burkholderia*, Secondary metabolites, Siderophores, *Colletotrichum gloeosporioides*, Post-harvest avocado fruits

## Abstract

**Abstract:**

Siderophores are iron-chelating secondary metabolites that can effectively control fungal diseases in several agronomically important crops. In the present study, the bacterium *Burkholderia contaminans* MSR2 was found to profusely accumulate siderophores when grown under iron-limiting conditions. Therefore, subsequent experimental procedures were performed to determine whether the siderophore-rich supernatants of *B. contaminans* MSR2 were effective against anthracnose disease caused by *Colletotrichum gloeosporioides* in post-harvest avocado fruits. Dual culture assays performed in vitro showed that *B. contaminans* MSR2 produced an approximate 25% reduction in the radial mycelial growth of *C. gloeosporioides*. Moreover, cell-free supernatants (CFSs) with a siderophore concentration of 58.08 mM deferoxamine mesylate equivalent caused a 78% reduction of *C. gloeosporioides* growth under in vitro conditions. In avocado fruits, siderophore-rich CFSs significantly reduced the severity and incidence of post-harvest anthracnose disease, similar to the effect produced by the Captan 4L fungicide (2-(trichloromethylsulfanyl)-3a,4,7,7a-tetrahydroisoindole-1,3-dione) at 2 g/L. Untargeted metabolomic analyses of *B. contaminans* CFSs obtained under iron-limited conditions revealed the predominant accumulation of the ornibactin C8 and pyochelin siderophores, in addition to numerous other antimicrobial compounds, including secondary siderophores and further biological compounds known to have fungitoxic activity. These results support the proposal that siderophore-rich bioactive CFSs from *B. contaminans* MSR2 can be effectively employed for biological control of post-harvest anthracnose in avocado fruits.

**Key points:**

• *B. contaminans MSR2 cell-free supernatants (CFSs) inhibited C. gloeosporioides’growth.*

• *CFSs and Captan similarly reduced post-harvest anthracnose damage in avocado fruits.*

• *Pyochelin, pyochelin-like, ornibactin C8, and tetrapeptide siderophores were prominent anti-anthracnose CFSs.*

**Supplementary information:**

The online version contains supplementary material available at 10.1007/s00253-026-13715-2.

## Introduction

Avocado (*Persea americana* Mill.) is a high-value crop cultivated worldwide in tropical and subtropical areas. Avocado’s elevated market value is due to its highly nutritious components, primarily vitamins and minerals; it also reflects its importance as a source of essential oils for the cosmetic industry (Bill et al. 2016). However, avocados are susceptible to several diseases that frequently result in extensive post-harvest losses during transportation and storage. The most relevant post-harvest avocado disease in tropical and subtropical regions of the world is anthracnose, which is caused by *Colletotrichum gloeosporioides* Penz.

﻿﻿﻿An﻿ epidemiological study designed to determine the pathogenicity of *Colletotrichum* spp. associated with avocado anthracnose in Israel reported, for instance, a 100% disease incidence for all isolates tested (Sharma et al. 2017).

Anthracn﻿ose ﻿is the main sanitary limitation in the avocado supply chain, causing a significantly reduced shelf life and lower marketability (Glowacz et al. 2017; Sharma et al. 2017). Anthracnose symptoms appear after harvest, usually when the fruits begin to ripen. The disease manifests itself as dark brown circular spots that develop on the avocado pericarp, in addition to other symptoms, such as softening and rotting of the mesocarp (Prusky et al. 2001; Damm et al. 2013).

Chemical fungicides are predominantly used for the control of anthracnose. For example, the copper-based fungicides captafol and benomyl are applied in the field at 14 to 28 days intervals from fruit formation to harvest. Subsequent post-harvest chemical treatments include immersion of the fruits in thiabendazole, at 1.5–2.0 g/L, and the use of prochloraz, at 0.5 g/L, in combination with protective waxes (Eckert and Ogawa 1985; Bill et al. 2014; Sivakumar and Bautista-Baños 2014). On the other hand, the increasing concern regarding the impact that chemical fungicides might have on the environment and on human health, combined with the demand for organically produced crops, has led to the research and development of alternative antifungal control methods, such as antagonistic microorganisms and their secondary metabolites. The use of microbial antagonists to control anthracnose in tropical fruits has been widely reported; however, their application for the protection of avocado fruits has been limited. For instance, applying naturally antagonistic yeast cultures on avocado fruits infected with *C. gloeosporioides* and *Colletotrichum acutatum* decreased the severity of the disease by 34 and 32%, respectively (Campos-Martínez et al. 2016). Also, the use of *Bacillus atrophaeus* cell-free supernatants (CFSs) increased the post-harvest viability of avocado and soursop by reducing anthracnose severity by 41%. Authors attributed this reduction to the lipopeptides present in the CFSs, such as surfactin, bacillomycin, and iturin (Guardado-Valdivia et al. 2018). Most recently, Granada et al. (2020) evaluated the protective effect of a bacterial extract from *Serratia* sp. against *C. gloeosporioides* in avocado fruits. They found a 63–79% reduction in wound size when they applied the bacterial extracts directly on previously infected fruits.

In this context, siderophore-producing rhizobacteria have provided promising results when used to control phytopathogenic fungi. Siderophores are low molecular weight compounds (500–1000 Da) that act as chelating agents with a high affinity for Fe^3+^. Siderophores are synthesized to maintain bacterial growth rate and metabolic activity when grown in iron-deficient conditions (Rocha et al. 2025). Moreover, the use of siderophore-rich bacterial CFSs has been shown to be effective in vitro against several phytopathogenic fungi, including *Botrytis cinerea*, *Rhizoctonia solani*, and *C. gloeosporioides* (Calvente et al. 2001; de los Santos-Villalobos et al. 2012; Gull 2012; Sasirekha and Srividya 2016). More recently, Lambrese et al. (2018) found that extracts of purified enterochelin siderophore isolated from the *Kosakonia radicincitans* bacteria reduced decay symptoms occasioned by *B. cinerea* in apple fruits. More recently, two siderophores, pyochelin and ornibactin present in *Burkholderia cenocepacia* culture supernatants, were found to synergistically suppress the growth of *Cercospora zeina*, an aggressive maize fungal pathogen, and to significantly reduce gray leaf spot disease symptoms in maize plants (Zheng et al. 2025). Congruent with this background, the main objective of the present study was to extend the knowledge of these highly promising biological control compounds by performing diverse assays to test their antagonistic effect vs. *C. gloeosporioides* MYA 456. The latter was approached using an experimental strategy that included (i) the determination of the effect produced by the siderophore-rich CFSs produced by *Burkholderia contaminans* MSR2 on *C. gloeosporioides* mycelial growth in vitro, (ii) the extent of anthracnose disease in infected avocado fruits during post-harvest storage, and (iii) an untargeted metabolomic analysis designed to identify possible bioactive biological compounds. The results obtained suggest that siderophores, and perhaps other secondary metabolites secreted by *B. contaminans* MSR2, contributed to the potent bioactive activity observed against this pernicious fungal pathogen in avocado fruits. They also indicated a greater protective effect vs. *C. gloeosporioides* avocado disease than the one reported in a previous study where *B. atrophaeus* and *Bacillus mycoides* were employed as bio-control agents (Guardado-Valdivia et al. 2018; Guerrero-Barajas et al. 2020). This property can potentially be employed for fungicide-free post-harvest protection schemes designed for avocado fruits and other commercially important fruit crops. Moreover, the findings of the present study shed light on the still fragmentary knowledge regarding the molecular mechanisms underlying the biocontrol capacity of *Burkholderia* strains against fungal diseases in avocado fruits.

## Materials and methods

### Bacteria strain preservation and pre-inoculum preparation

*B. contaminans* MSR2 was isolated from the rhizosphere of maize plants grown in central México. The molecular identity of the isolate and its antagonistic activity against maize phytopathogenic *Fusarium* spp. was previously described by Barrera-Galicia et al. (2021). Pure cultures of this strain were stored on Reasoner’s 2 A (R2A) agar plates (Difco Laboratories, Franklin Lakes, NJ, USA) at 4 °C for 30 days, or at −70 °C in 20% glycerol, for longer periods. The strain *B. contaminans* MSR2 is available upon request from the Laboratorio de Fisiología de la Defensa de Plantas, Cinvestav-Irapuato, México. Before the bioassays, a pre-inoculum of the bacterial strain was prepared in 10 mL of nutrient broth. After 24 h of incubation at 150 rpm and 28 °C, 1-mL aliquots were centrifuged at 5000 rpm for 5 min. The pellets were suspended in a sterile 0.85% NaCl solution to obtain a cell density equivalent to an OD_595_ of 0.2. The bacterial inoculum was always prepared fresh before use.

### Phytopathogenic fungal strain culture conditions

A conidia suspension of the phytopathogenic *C. gloeosporioides* strain ATCC MYA 456 was prepared from mature fungal cultures grown on potato dextrose agar (PDA) for 10 days. Conidia were harvested in Triton 0.01%, and the working conidial suspensions were adjusted to a concentration of 1 × 10^6^ spores mL^−1^.

### Detection of bacterial siderophores

Siderophore production was determined qualitatively using chrome azurol sulfonate (CAS) agar medium (Alexander and Zuberer 1991). To prepare 1 L of CAS agar, 60.5 mg CAS was dissolved in 50 mL of water and mixed with 10 mL of a 1 mM FeCl_3_.6H_2_O solution, previously dissolved in 10 mM HCl. This solution was slowly added, while stirring, to 1.82 mg of hexadecyltrimethyl ammonium dissolved in 40 mL water; the resulting solution was autoclaved. A mixture of 750 mL H_2_O, 0.3 g of KH_2_PO_4_, 0.5 g of NaCl, 1 g of NH_4_Cl, 30.24 g PIPES buffer, and 15 g of agar was adjusted to pH 6.8 with NaOH and autoclaved. A third solution was prepared containing 2.0 g glucose, 2.0 g mannitol, 493 mg MgSO_4_·7H_2_O, 11 mg CaCl_2_, 1.17 mg MnSO_4_·H_2_O, 1.4 mg H_3_BO_3_, 0.004 mg CuSO_4_·5H_2_O, 1.2 mg ZnSO_4_·7H_2_O, and 1.0 mg Na_2_MoO_4_·2H_2_O in 70 mL H_2_O and autoclaved. After cooling to 50 °C, all three solutions were slowly mixed to avoid bubble formation. Finally, 30 mL of filtered casamino acids (10% w/v) were sterilized by filtration using a 0.22-µm pore-size cellulose membrane (Millex GV; Cork, Ireland) and were added to the main mixture. Twenty-microliter aliquots of bacterial inoculum were spotted on the CAS agar plates and incubated at 28 °C for 72 h. The formation of an orange halo around the bacterial colonies evidenced siderophore production.

### Colorimetric siderophore quantification

Siderophore quantification in the CFSs was performed using the chrome azurol S (CAS) assay as modified by Alexander and Zuberer (1991). Briefly, 100 µL of *B. contaminans* MSR2 pre-inoculum was cultured in 50-mL iron-free M9 minimal medium. After 96-h incubation at 28 °C, siderophore concentration was measured by mixing 500 μL of modified CAS assay solution with 500 μL of bacterial-filtered supernatant and 10 μL of 0.2 M 5-sulfosalicylic acid, which was used as a shuttle solution to facilitate iron exchange during the CAS reaction, was added to each well of a 96-well microtiter plate. After a 60-min stabilization period, the absorbance of the solutions was measured at 630 nm in a microplate reader (Bio-Rad, Hercules, CA, USA). A standard calibration curve was made using deferoxamine mesylate as standard and M9 medium as a reference solution containing no siderophores. Siderophore concentration in the samples was expressed as μM of deferoxamine mesylate equivalent (μM DFOM_eq_). All measurements were made in three independent biological replicates performed.

### In vitro evaluation of antagonistic activity

A dual culture assay (Zivkovic et al. 2010) was used to determine the antagonistic effect of *B. contaminans* MSR2 against *C. gloeosporioides* MYA 456. Twenty-microliter aliquots of bacterial pre-inoculum were placed as a single horizontal streak on PDA agar, and a fungal plug (7 mm in diameter) was placed 4.2 cm away from the bacterial streak on the opposite side of each plate. Each confrontation was carried out using six independent plates. The control treatments consisted of plates inoculated with *C. gloeosporioides* only. Radial growth of the mycelia was measured 10 days after incubation at 28 °C using ImageJ, version 1.52a software (Abràmoff et al. 2004; Schneider et al. 2012). The antagonistic activity was expressed as the percentage reduction of the fungal colony’s diameter relative to the control treatment calculated as [(D_control_)−D_treatment_)/D_control_] × 100.

### Cell-free supernatant preparation for inhibitory bioassays

The bacteria strain was cultured under two different conditions to test the siderophore’s role in the inhibitory capacity of *B. contaminans* MSR2. Siderophore-rich CFSs were produced from bacterial cultures incubated at 28 °C for 96 h in M9 iron-free, [Fe0], medium. A set of siderophore-depleted CFSs was produced by adding FeCl_3_ 100 μM, [Fe100], to the M9 medium. Inoculated media were incubated in a rotatory shaker (150 rpm) for 96 h at 28 °C. Three biological replicates were prepared, using uninoculated media as a negative control.

After incubation, the cultures were centrifuged at 10,000 rpm for 20 min, and the supernatants were filtered twice, first with a 0.45-μm nylon syringe filter (Nalgene, Rochester, NY, USA) and subsequently using 0.22-μm polyvinylidene fluoride membranes (Millex GV). The supernatants were collected in sterile plastic tubes and stored at 4 °C until their use in the bioassays.

### In vitro evaluation of bacterial supernatants against a phytopathogenic fungal strain

To determine the inhibitory effects of siderophore-rich CFSs, *C. gloeosporioides* was grown together with contrasting *B. contaminans* CFSs. For this purpose, PDA-CFS agar was prepared using either siderophore-rich CFSs, i.e., CFS [Fe0], or CFSs having no siderophore activity, i.e., CFS [Fe100], which were generated as mentioned above. Briefly, molten PDA was mixed with bacterial CFSs in a 1:1 *v*/*v* ratio. When the media solidified, 10 μL of a *C. gloeosporioides* MYA 456 conidial suspension was placed in the middle of the Petri dishes. Subsequently, the inoculated plates were incubated at 28 °C for 10 days. The experiment was repeated thrice, each including three biological replicates per treatment.

### Determination of siderophore´s effective concentration (EC_50_)

To determine the concentration of the *B. contaminans* MSR2 siderophore mixture capable of inhibiting 50% of fungal mycelial growth, different CFSs with known siderophore concentrations, i.e., 5, 20, 40, and 60 μM DFOM_eq_, were homogenously mixed with molten sterile PDA agar. Once the medium was solidified, 10 μL of *C. gloeosporioides* MYA 456 conidial suspensions were placed in the middle of Petri dishes. The control treatment consisted of PDA plates without bacterial CFSs. Plates were incubated at 28 °C for 10 days. Each treatment was repeated three times, using five independent Petri dishes per replicate, and radial mycelial growth was measured using ImageJ software.

### In vivo antifungal assay of Burkholderia CFSs

Freshly harvested avocado fruits (*P. americana* Mill. var. Hass) were used for in vivo antifungal assays. The fruits were surface sterilized by dipping them in 0.01% sodium hypochlorite solution for 5 min. They were rinsed with sterile distilled water and air-dried in a laminar flow cabinet. Three uniform wounds, 10 mm × 3 mm, were made with a sterile scalpel on the pericarp of each avocado fruit (four fruits per treatment). Subsequently, the fruits were exposed to different treatments: (i) CFSs with 60 μM DFOM_eq_ siderophore activity, (ii) CFSs without siderophore activity, (iii) sterile distilled water (untreated control), and (iv) a 2 g/L commercial antifungal formulation (Captan 4L [2-(trichloromethylsulfanyl)−3a,4,7,7a-tetrahydroisoindole-1,3-dione]; Drexel Chemical Co., Memphis, TN, USA; positive control). For fungal inoculation, 15 μL of *C. gloeosporioides* spore suspension was placed in each wound. Afterward, avocado fruits were incubated in sterile plastic containers at 28 °C and 95% relative humidity for 10 days. Disease incidence, defined as the percentage area of infected fruit, and severity, measured as the lesion diameter in millimeters (mm), were determined using ImageJ. Three independent replicates of this experiment were performed.

### Liquid chromatography (LC)–tandem mass spectrometry (MS)/MS) untargeted analysis of bacterial siderophore-rich CFSs having antifungal activity

The analysis of siderophore production in the CFSs obtained from 48-h cultures of *B. contaminans* MSR2 in M9 iron-free medium and M9 complemented with 100 μM of FeCl_3_ was performed using LC-MS/MS data provided as a service by Novogene Corporation Inc. (Sacramento, CA, USA). For each condition, three independent CFS samples were processed and analyzed. Sample processing for LC-MS/MS started with 100 mL of CFSs, which were filtered through 0.2-μm polytetrafluoroethylene (PTFE) membranes (Agilent Technologies, Santa Clara, CA, USA) and injected into a 300-mg MaxiClean C_18_ solid-phase extraction cartridge (Alltech Associates Inc., Deerfield, IL, USA) following the manufacturer’s recommendations. The cartridges were rinsed with deionized water and 50% methanol, respectively, and the siderophore fraction was subsequently eluted with 100% methanol as instructed by Martin et al. (2006). Purified siderophore fractions were then injected into a UHPLC system (ExionLC AD, SCIEX, Foster City, CA, USA) coupled to a TripleTOF 6600+ mass spectrometer (AB SCIEX, Foster City, CA, USA) as described by the service provider. Chromatographic separations were optimized to maximize metabolome coverage. The first separation was performed using an ACQUITY UPLC HSS T3 column (1.8 µm × 2.1 mm 100 mm, Waters, Milford, MA, USA) maintained at 40 °C. The mobile phases consisted of ultrapure water with 0.1% formic acid (phase A) and acetonitrile with 0.1% formic acid (phase B), at a flow rate of 0.40 mL/min and an injection volume of 4 µL. The elution gradient started at 5% B, ramped to 99% B at 6.0 min, held at this concentration for 1.5 min, and returned to the initial conditions in 7.6 min, for a total run time of 10 min. Subsequently, a complementary separation was conducted using a ACQUITY Premier BEH Amide column (1.7 µm × 2.1 mm × 150 mm, Waters) maintained at 40 °C. The mobile phases consisted of 60% acetonitrile, 30% water, and 10% methanol with 20 mM ammonium formate (pH 10.6) for phase A and 40% acetonitrile, 60% water with 20 mM ammonium formate (pH 10.6) for phase B, at a flow rate of 0.40 mL/min. The gradient began at 95% A, decreased to 5% A by 5.5 min, was maintained for 1 min, and returned to the initial conditions at 6.51 min, for a total run time of 10 min.

Mass spectra were acquired within a mass range of 50 to 1000 m/z in positive ionization mode. The electrospray (ESI) source parameters were as follows: ion spray voltage, 5000 V; source temperature, 550 °C; ion source gas 1, 50 psi; ion source gas 2, 60 psi. MS1 acquisition was performed with a collision energy of 10 V, while MS2 data acquisition was performed in data-dependent acquisition (DDA) mode, targeting the most intense precursor ions in each full scan. Spectra were acquired using beam-type collision-induced dissociation (CID) with nitrogen as collision gas, applying variable collision energies (30–15 V). Mass calibration was performed using an internal standard to ensure mass accuracy throughout the runs.

### Metabolomic data analysis

The original data file acquired by LC-MS was converted to mzML format using ProteoWizard (Chambers et al. 2012). Peak detection, alignment, and retention time correction were performed using MS-DIAL version 5.5 (Tsugawa et al. 2015). Alignment results were downloaded as a comma-separated value (CSV) file and processed in R studio software (Posit 2023) for filtering. Peaks with a missing rate higher than 50% within each group of samples were excluded. The processed data matrix was used for subsequent univariate and multivariate analyses. Data were normalized by auto-scale, mean-centered, and divided by the standard deviation of each variable using MetaboAnalyst 6.0 (Pang et al. 2024).

Univariate analyses, including *t-*tests and volcano plots, were conducted to identify significant differences between the two culture conditions in which *B. contaminans* MSR2 strain growth was performed: [Fe0] and [Fe100]. Supervised multivariate methods, such as partial least squares discriminant analysis (PLS-DA) and hierarchical cluster analysis (HCA), were used to explore sample clustering. The discriminant features putatively annotated based on MS/MS spectra were used to determine metabolite classes and metabolic pathways significantly affected under the experimental conditions. Pathway enrichment analysis was conducted using MetaboAnalyst 6.0, based on the Kyoto Encyclopedia of Genes and Genomes (KEGG) database (Kanehisha and Goto 2000), using the pathway library reference metabolome of *Burkholderia mallei* ATCC 23344 (bma, KEGG organisms abbreviation).

### Metabolite annotation

MS2 spectral deconvolution was performed using MS-DIAL 5.5 default parameters. Mass spectra were then compared with those previously detected in members of the *Burkholderia* genus (refer to Table S1 in Supplementary material)*.* The putative annotation of *B. contaminans*’ MSR2 siderophores was obtained by comparing their experimental mass spectra with published mass spectral data using the MONA and GNPS databases (Hilbig et al. 2013; Wang et al. 2016), including entries corresponding to pyochelin (Moree et al. 2012) and ornibactin C8 (Stephan et al. 1993).

### Statistical analysis

The differences among treatments in each experiment were compared using one-way analyses of variance (ANOVA), followed by a Tukey post-hoc test (*p* < 0.05). All statistical analyses were performed using R software (R Core Team 2023) through the RStudio integrated development environment (Posit 2023).

## Results

### Siderophore detection and colorimetric quantification

*B. contaminans* MSR2 bacteria grown in CAS agar media produced the characteristic orange haloes around their respective colonies, which were indicative of positive siderophore production (Supplemental Fig. S1). After a 96-h culture period in M9 free iron medium, the siderophore concentration in the CFS reached 72.6 ± 3.9 μM DFOM_eq_ according to the modified CAS assay solution method employed.

### Antagonistic activity toward *C. gloeosporioides* growth in *B. contaminans* MSR2 CFSs

**Fig. 1:**
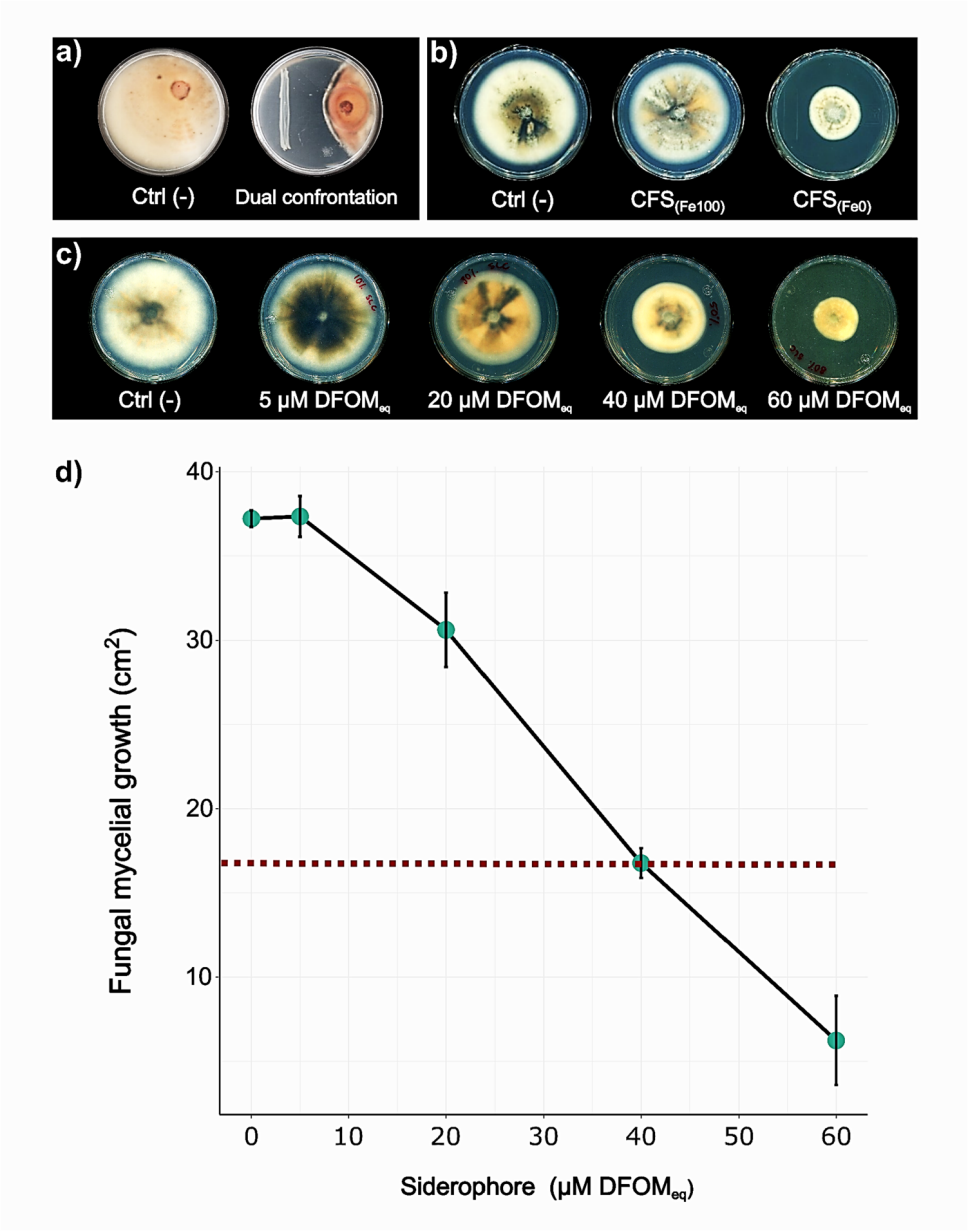
Secreted metabolites from *B. contaminans* MSR2 grown in iron-limiting conditions inhibit the growth of *C. gloeosporioides*. **a** Radial mycelial growth of *C. gloeosporioides* in PDA medium (left), and in confrontation with *B. contaminans* MSR2 growing in PDA medium (right); **b** radial growth of *C. gloeosporioides* in PDA combined with M9 media only (Ctl-), in PDA combined with *B. contaminans* MSR2 CFSs lacking siderophores (CFS [Fe100]), and in PDA combined with siderophore-rich *B. contaminans* MSR2 CFSs (CFS [Fe0]); **c** in vitro assay showing the siderophore dose, provided by CFS [Fe0], required to reduce mycelial growth by half (red, punctuated line in panel **d**) as determined by the inverse relationship observed between increased siderophore concentration and reduced fungal mycelial growth

Dual confrontation assays showed that *B. contaminans* MSR2 exhibited a 24.5% ± 2.1 (*n* = 3) inhibitory effect on the mycelial growth of *C. gloeosporioides* (Fig. 1a). Further in vitro inhibition assays were conducted with *B. contaminans* MSR2 to determine if the inhibitory effect observed could be attributed to the secreted siderophores. Thus, no inhibition, compared to the control, was observed when siderophore-depleted CFSs, i.e., CFS [Fe100], were used, and this difference was not statistically significant (*p* > 0.05). In contrast, supplementation assays using PDA media combined with siderophore-rich CFSs, i.e., CFS [Fe0], resulted in a significant inhibition of *C. gloeosporioides* mycelial growth (69.7% ± 1.4; *p* < 0.05; Fig. 1b). These results strongly suggest that siderophores were required for the observed in vitro inhibition of *C. gloeosporioides* mycelial growth.

### EC_50_ of siderophore-rich CFS from *B. contaminans* MSR2 against fungal mycelial growth

The EC_50_ assay, performed by supplementing PDA agar with varying amounts of CFS [Fe0] from *B. contaminans* MSR2, showed an inverse *C. gloeosporioides* mycelial growth rate in response to increasing siderophore concentrations in the bacterial CFS [Fe0] (Fig. 1c). After 10 days of incubation, the effective dose required to reduce mycelial growth by half was 40 μM DFOM_eq_. Furthermore, data in Fig. 1d indicate that the minimal fungal radial growth (6.2 ± 2.7 cm^2^) was reached at a siderophore concentration of 60 μM DFOM_eq_. Significant differences were found between all treatments (*p* < 0.05).

### In vivo antagonistic activity against *C. gloeosporioides* in *B. contaminans* MSR2 CFSs

**Fig. 2:**
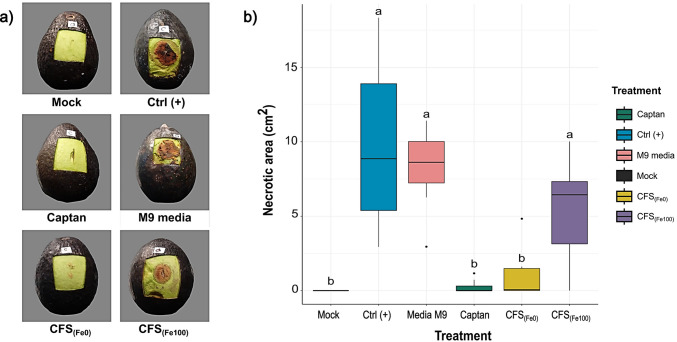
Effect of siderophore-rich *B. contaminans* MSR2 cell-free supernatants (CFSs) on anthracnose disease development in avocado fruits. **a** Images show partially wounded avocado fruits submitted to different treatments: uninfected control (Mock); infected with *C. gloeosporioides* spores (Ctrl^+^); sprayed with a commercial fungicide (Captan, 2 g/L); sprayed with bacterial culture media (M9 media) or with siderophore-rich (34 μM DFOM, CFS [Fe0]) or siderophore-depleted cell-free supernatants (CFS [Fe100]), prior to fungal infection; **b** the boxplots indicate the necrotic area, in cm^2^, produced in response to infection with a conidial suspension of *C. gloeosporioides* in avocado fruits subjected to different treatments. Avocados were treated with either M9 medium (M9), a commercial fungicide (Captan), siderophore-rich (CFS [Fe0]), or siderophore-depleted (CFS [Fe100]) cell-free supernatants obtained from *B. contaminans* MSR2, before fungal infection. Both negative (Mock) and positive (Ctrl+) infection controls were also included. Smaller necrotic areas are indicative of higher anthracnose disease suppression. Different letters over the boxplots indicate significantly different levels of anthracnose suppression at *p* ≤ 0.05 (one-way ANOVA, Tukey-Kramer test, *n* = 8)

This study proposes that siderophore-rich extracts produced by *B. contaminans* MSR2 represent a potential alternative for the efficient control of anthracnose disease in avocado fruits. The results shown in Fig. 2a indicate that untreated avocado fruits and avocado fruits sprayed with M9 media only were readily infected with a conidial suspension of *C. gloeosporioides* 10 days post-inoculation (Ctrl (+) and M9 media panels). In contrast, the application of an aliquot taken from a siderophore-rich extract from *B. contaminans* MSR2 visibly suppressed anthracnose disease and significantly reduced infection severity (CFS [Fe0] panel). The protective effect conferred by the CFS [Fe0] treatment was similar to the one produced by the application of the Captan 4L fungicide (Captan panel), whereas the application of CFS [Fe100] extracts on *C. gloeosporioides*–infected wounds displayed weaker infection symptoms than the positive control group (compare CFS [100] and Ctrl (+) panels). The boxplots shown in Fig. 2b confirmed that the siderophore-enriched treatment significantly reduced the size of the necrotic lesions observed on their surface (CFS [Fe0]; 0.95 ± 1.6 cm^2^) compared to those produced in infected controls (Ctrl+; 9.8 ± 5.7 cm^2^). Furthermore, the protective effect conferred by the CFS [Fe0] treatment was similar to that produced by the application of the commercial fungicide (Captan; mean lesion area of 0.28 ± 0.4 cm^2^). Although the application of siderophore-depleted extracts (CFS [Fe100]) displayed weaker *C. gloeosporioides*–infected infection symptoms than the Ctrl (+) and M9 treatments (refer to Fig. 2a), the apparent protection provided, in terms of the extent of the necrotic areas produced, was found not to be significantly different. However, the weaker infection symptoms observed in CFS [Fe100]–treated avocado fruits suggest that secondary metabolites other than siderophores, e.g., glidobactin and others (see below), could contribute to the in vivo anthracnose suppression produced by *B. contaminans* MSR2 CFSs.

### Untargeted metabolome profile of antifungal siderophore-rich supernatants produced by *B. contaminans* MSR2

**Fig. 3:**
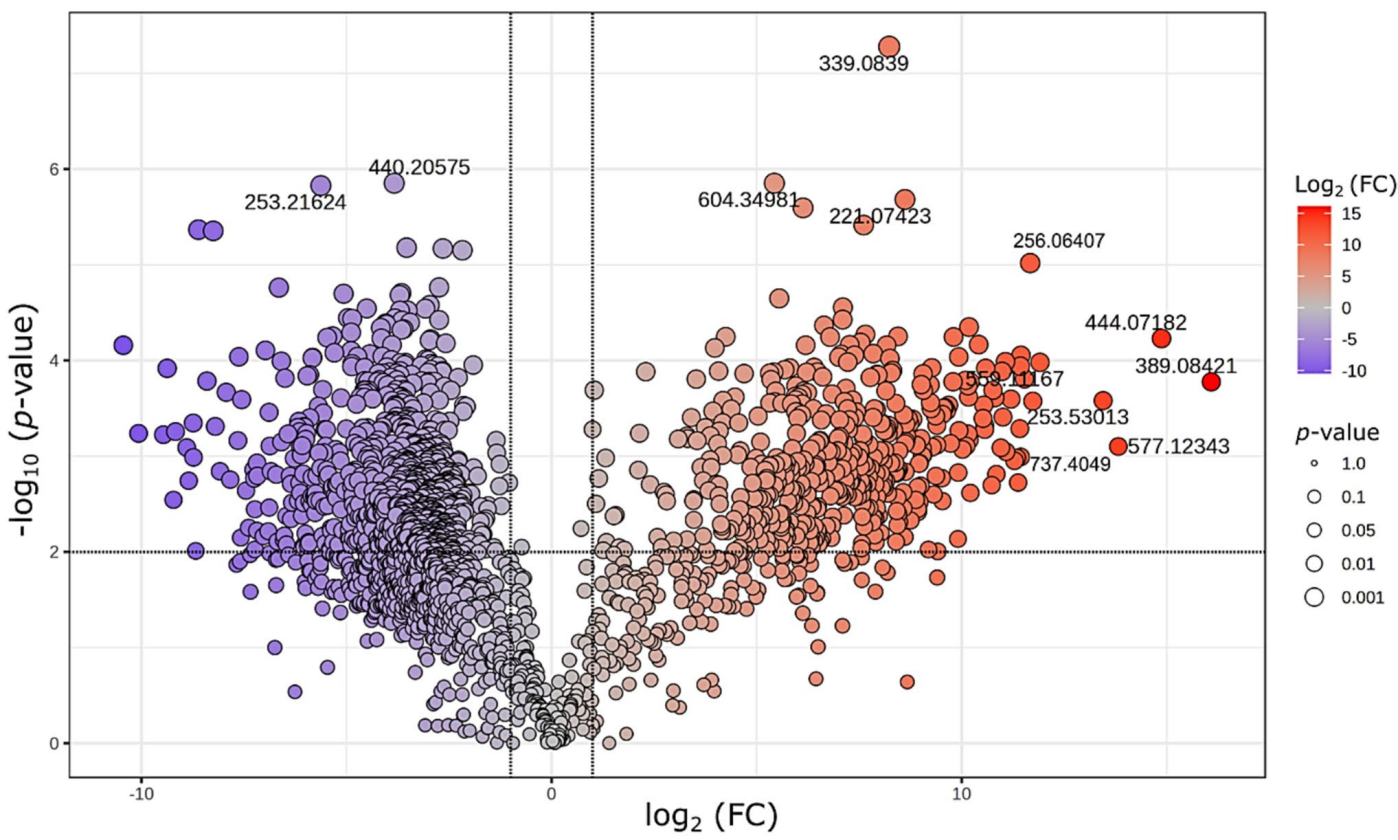
Volcano plot of differentially abundant metabolites produced by *B. contaminans* MSR2 under iron-depleted [Fe0] and iron-supplemented [Fe100] conditions. Metabolites significantly upregulated in the siderophore-enriched, [Fe0] condition appear in red (right side), while those downregulated (more abundant in the siderophore-depleted, [Fe100] condition) are shown in blue (left side)

The total ion chromatograms (TIC) obtained from the analysis of *B. contaminans* MSR2 CFSs by LC-MS/MS showed great complexity and a differential metabolite profile, which probably arose from multiple other compounds, in addition to siderophores, found in the CFSs [Fe0] even after a solid-phase extraction pre-purification step (Supplemental Fig. S2). Once the data was filtered to eliminate background noise, a 2000 feature matrix was generated from which the differential metabolites between both culture conditions, i.e., siderophore-depleted, [Fe100], and siderophore-rich, [Fe0], were identified using a univariant statistical analysis. The volcano plot thus generated (Fig. 3) shows the distribution of *m/z* signals in relation to their relative abundance (fold-change, [FC] ≥ 2) and statistical significance (*p* ≤ 0.05). The above exercise allowed the identification of 620 and 1230 overexpressed and repressed features under [Fe0] and [Fe100] conditions, respectively. The most significant *m/z* signals detected in the bioactive CFSs derived from *B. contaminans* MSR2 bacteria cultured under [Fe0] conditions were the following: 389.08421, 444.07182, 577.12343, 253.53013, 325.04956, and 737.4049.

**Fig. 4:**
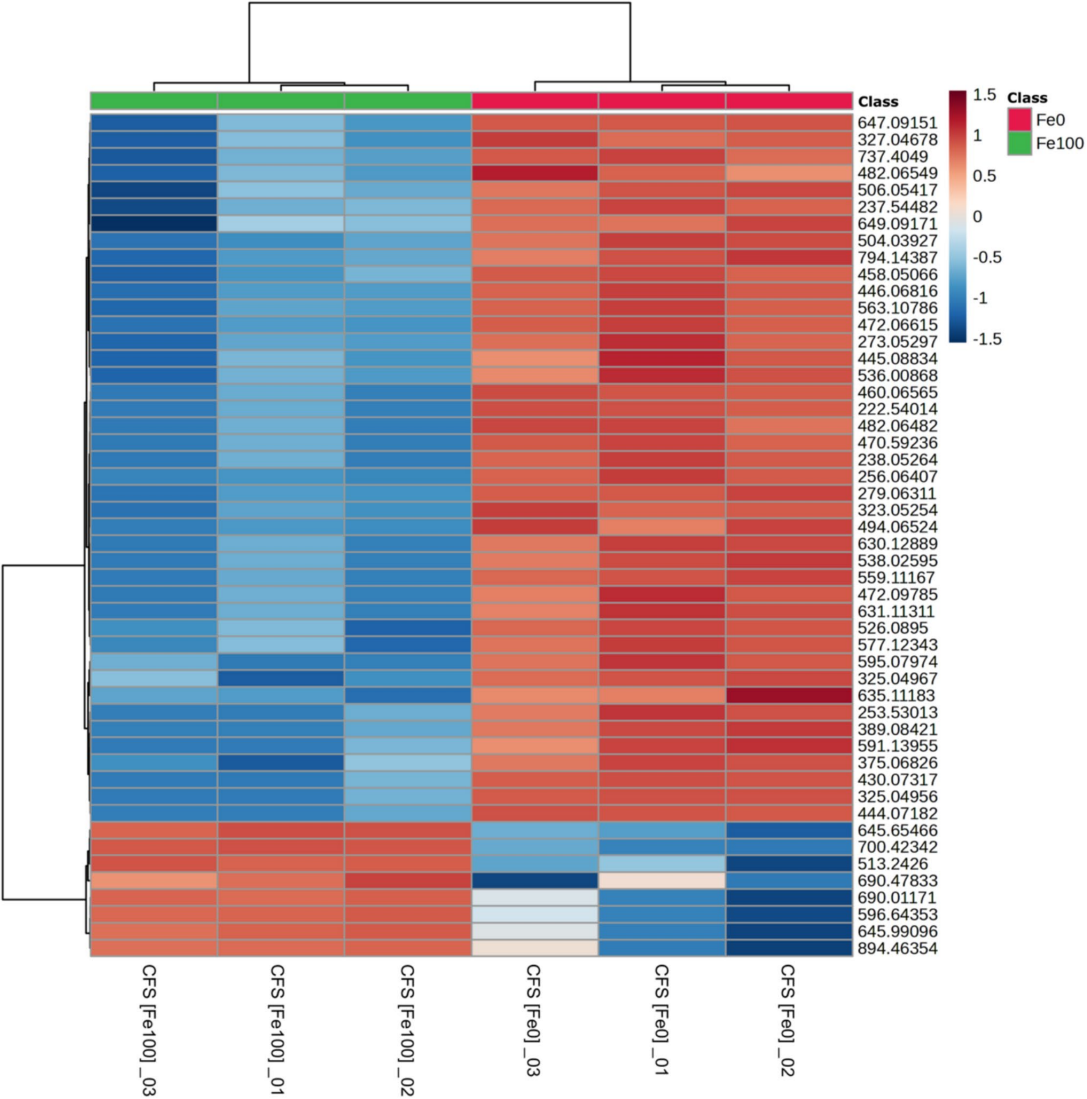
Heatmap of differentially abundant metabolite ions detected in the CFSs of *B. contaminans* MSR2 cultured under iron-supplemented (CFS [Fe100]; green bar) and iron-deficient (CFS [Fe0]; bright red bar) conditions. The figure shows the normalized intensities of the selected signals derived from three independent biological replicates. The color scale shown in the upper right section is an indication of the relative abundance of each peak where red and blue are indicative of high and low abundance, respectively

In order to observe the differences in the metabolic composition of the *B. contaminans* CFSs generated under [Fe0] and [Fe100] conditions, a hierarchical clustering analysis of the most discriminating ions was performed, based on the PLS-DA model. The relative abundance of the 50 ion signals selected per culturing condition based on Variable Importance in Projection (VIP) scores greater than 1 is shown in the heat map in Fig. 4. Here, the formation of two well-defined groups is observed, where the highest abundance of metabolites is clearly found in the CFSs generated by *B. contaminans* MSR2 bacteria cultured under siderophore-inducing [Fe0] conditions. The list of the candidate metabolites potentially present in the CFS of *B. contaminans* MSR2 CFS [Fe0] is included in Table S2 in the Supplementary Material section. The putative annotation was based on exact mass values from MS1 data.

**Fig. 5:**
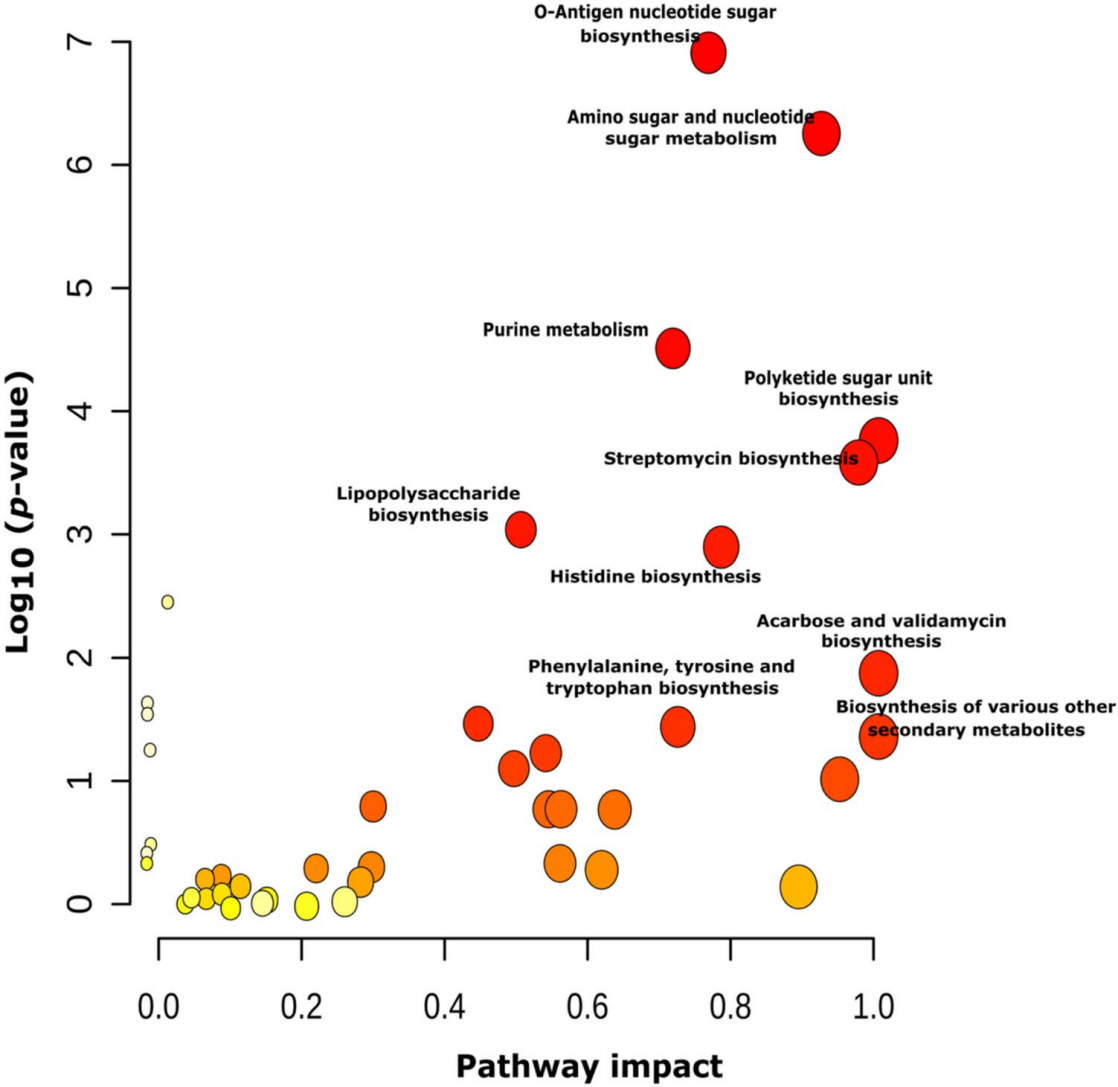
Metabolic pathway impact of differentially accumulated metabolites (DAMs) overexpressed under siderophore-enriched [Fe0] conditions. Metabolic pathways that were significantly induced under this condition are presented as circles, whose color and size were determined by their statistical significance (y-axis) and pathway impact value/enrichment factor (x-axis), respectively

Likewise, a functional enrichment analysis was carried out using the differentially accumulated metabolites (DAMs) overexpressed under [Fe0] conditions. These features were selected based on a fold-change threshold ≥ 2 (Fig. 5). To determine the metabolic pathways most significantly associated with the compounds produced by *B. contaminans* MSR2 in response to iron deprived, [Fe0] conditions, a pathway topology analysis was subsequently performed. As shown in Fig. 5 and Supplemental Table S3, particularly relevant metabolic pathways were those corresponding to primary metabolism (O-antigen nucleotide sugar biosynthesis; amino sugar and nucleotide sugar, purine and histidine metabolism) all showing significant *p*-values ≤ 0.001. In addition, several pathways associated with the biosynthesis of bacterial secondary metabolites (i.e., polyketide sugar unit biosynthesis, streptomycin biosynthesis, acarbose, validamycin) and other secondary metabolites were also enriched. The paerucumarin biosynthetic pathway and phenylalanine, tyrosine, and tryptophan biosynthesis were of salient importance (Supplemental Figs. S3 and S4), the latter due to its direct relationship with siderophore biosynthesis via the non-ribosomal peptide synthetase (NRPS) multienzyme-dependent pathway.

**Fig. 6:**
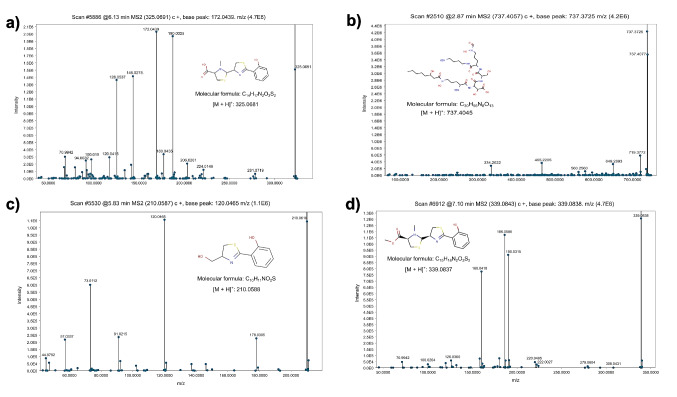
The mass fragmentation spectra of the predominant bioactive compounds detected in the CFSs of *B. contaminans* MSR2 grown under siderophore-enriched [Fe0] conditions. The mass spectra shown correspond to **a** pyochelin (C_14_H_16_N_2_O_3_S_2_; 325.0691 m/z), **b** ornibactin C (C_30_H_56_N_8_O_13_; 737.4063 m/z), **c** auregine (C_10_H_11_NO_2_S, 209.05105 m/z), and **d** pyochelin methyl ester (C_15_H_18_N_2_O_3_S_2,_ 337.0837 m/z)

For a more accurate putative annotation, the fragmentation patterns of the most abundant signals were compared with data from GNPS base libraries. This led to the annotation of the pyochelin (retention time [RT] of 6.13 min and 325.0691 m/z [M^+^H]^+^), pyochelin methyl ester (RT of 5.66 min and 339.0466 m*/*z [M^+^H]^+^), and ornibactin C8 (RT of 2.91 min and 737.4063 m/z [M^+^H]^+^) siderophores and of the thiazoline antibiotic aerugine (RT of 5.77 min and 210.0585 m/z [M^+^H]^+^), a byproduct of pyochelin and enantio-pyochelin biosynthesis, in the siderophore-enriched [Fe0] CFSs only. The fragmentation mass spectra of three of these metabolites are shown in Fig. 6. Notably, no MS2 library matches were found for some of the most abundant ions observed in the TICs obtained from the siderophore-enriched [Fe0] CFSs (Supplemental Fig. S2), including those with *m/z* = 361.2093, 472.0669, 504.0392, and 464.0560.

## Discussion

This study showed that the siderophore-rich CFSs produced during the culture of *B. contaminans* MSR2 under iron-limited conditions strongly inhibited post-harvest storage infection of avocado fruits by *C. gloeosporioides*. Pyochelin and ornibactin C8 were the major siderophores putatively annotated in the bacterial CFSs, based on MS/MS spectra comparisons. These findings agreed with a previous study reported by de los Santos-Villalobos et al. (2012) who successfully employed siderophore-abundant CFSs obtained from *Burkholderia cepacia* to inhibit the mycelial growth of *C. gloeosporioides* in vitro*.* The maximum growth inhibition reported in that study, i.e., 91%, was within the same high-inhibition range as the 78.2% obtained in the present work. The fungal growth–inhibitory effect was equivalent to that obtained by using the Captan 4L fungicide. They also resembled the drastic reduction of anthracnose rot observed in avocado fruits treated with thyme essential oils mixed with weakened versions of the widely employed, but toxic, Prochloraz anthracnose–controlling fungicide (Bill et al. 2016; Obianom and Sivakumar 2018). In addition, they were congruent with a number of studies indicating the potential use of *B. contaminans* for the biocontrol of other phytopathogenic fungi in commercial crops, including *B. cinerea*, in strawberry (Wang et al. 2018), *R. solani*, in maize (Tagele et al. 2018), *Macrophomina phaseolina*, in jute (*Corchorus olitorius*) (Zaman et al. 2021), *Fusarium* wilt in tomato (Heo et al. 2022), and post-harvest gray mold in grape and strawberry fruits (Shi and Du 2023).

However, in some of these cases, the protective effect was found not to be exclusively due to the bacterial CFSs (Wang et al. 2018), which suggests that not all the bioactive activity associated with the control of phytopathogens by *B. contaminans* is generated by secreted bioactive metabolites. This possibility needs to be experimentally evaluated in greater detail.

Regarding the above, the production of antimicrobial secondary metabolites having biocidal/biostatic activity has been proposed as the main mechanism deployed by *Burkholderia* to antagonize plant pathogens. They conform a group of chemically diverse compounds such as bacteriocin peptides, macrolides, N-containing heterocycles, non-ribosomal peptide-polyketide hybrid compounds, polyenes, polyynes, quinolones, siderophores, volatile organic compounds, and various others (Bach et al. 2022; Rodríguez-Cisneros et al. 2023). Their protective capacity is believed to stem from their capacity to disrupt either of various key physiological and biochemical processes in fungi associated with membrane and cell wall structure, electron transport networks, mitochondrial ATP/ADP transport, protein synthesis, and/or cell division (Yusuf et al. 2026). Siderophores in particular have been proposed to restrict the growth of plant pathogens by using their very high affinity for ferric iron in order to form strong ferric ion–siderophore complexes that lead to iron starvation conditions that restrict the growth of phytopathogenic bacteria and fungi, and sometimes prevent the germination of fungal spores (O'Sullivan and O'Gara 1992; Tracanna et al. 2021). Other workers have shown that siderophore toxicity to microorganisms, e.g., pyochelin, is caused by generating reactive oxygen species rather than by iron competition (Adler et al. 2012). Likewise, evidence indicating that siderophores may indirectly contribute to reinforce plant resistance vs. phytopathogens via a phenomenon known as nutritional immunity has also been reported. This particular event occurs through the activation of a variety of defense response mechanisms, such as cell wall lignification, accumulation of antimicrobial phenolic acids and flavonoids, callose deposition, flagellin-mediated signaling, and/or a jasmonic acid/ethylene-regulated induced systemic resistance event (Aznar et al. 2014: Wang et al. 2018; Heo et al. 2022; Platre et al. 2022). In this respect, the mass spectra obtained from the thorough analysis of the CFSs secreted by *B. contaminans* MSR2 under iron-limited conditions revealed the presence of the molecular ions having fragmentation patterns that corresponded to the pyochelin (C_14_H_16_N_2_O_3_S_2_), pyochelin methyl ester (C_15_H_18_N_2_0_3_S_2_), and ornibactin C8 (C_30_H_56_N_8_O_13_) siderophores as well as to the auregine (C_10_H_11_NO_2_S) thiazoline derivative, respectively. Ornibactin C8 and pyochelin are synthesized via the NRPS pathway and are widely distributed in *B. contaminans* and other species belonging to the *B. cepacia* clade (Bcc) (Deng et al. 2017; Esmaeel et al. 2019, 2020). Additional data suggested the presence of other antifungal-bioactive metabolites previously reported in *B. contaminans* and other Bcc members, such as the malleobactin and cepaciacheline siderophores (Schellenberg et al. 2007; Deng et al. 2017; Rodríguez-Cisneros et al. 2023; Yang et al. 2023) in addition to paerucumarin, a tyrosine-derived isonitrile metabolite first detected in *Pseudomonas aeruginosa* also known to chelate iron, promote biofilm formation, and increase antibiotic resistance (Qaisar et al. 2016; Iftikhar et al. 2020; Jeong et al. 2024). However, further research is needed to confirm the identity of several other bioactive metabolites presumably present in the CFSs of *B. contaminans* MSR2 cultured under siderophore-enriched [Fe0] conditions (see above). In this context, potential candidates, based on data presented in Fig. 5, are α-glucosidase inhibitors similar to acarbose analogues detected in *Actinoplanes* sp., *Bacillus stearothermophilus* and other bacteria (Zhang et al. 2024), validamycin-like antibiotics, similar to those synthesized by *Streptomyces* spp. (Li et al. 2019) and to gladiostatin, a novel natural compound identified in *Burkholderia gladiolis* that shares structural similarities with *Streptomyces* secondary metabolites (Nakou et al. 2020), glycosylated polyketides, similar to actinomycete-synthesized aromatic polyketides that contain deoxysugars as key structural elements (Hertweck et al. 2007), and di-rhamnolipid biosurfactants, resembling those generated by *Burkholderia pseudomallei* and *Burkholderia thailandensis* (Rodríguez-Cisneros et al. 2023). Other possibilities comprise a number of N-containing compounds like quinolinium iodides, quinolines, pyrazoles, pyrroles, and pyrrolidine derivatives, which were detected in in vitro confrontation assays showing a strong of mycelial growth inhibition of C. *gloeosporioides* and several other commercially important phytopathogenic fungi by *Burkholderia ubonensis* and *Burkholderia ambifaria* (Rivas-Guardado, personal communication). Nonetheless, the proposed protection against anthracnose in avocado fruits during post-harvest storage presumably provided by pyochelin resembled the demonstrated capacity shown by *P. aeruginosa* 7NSK2 to induce resistance to *B. cinerea* infection and *Pythium*-induced damping-off in tomato utilizing this and related secondary metabolites, such as salicylic acid, pyoverdin, and pyocyanin (Buysens et al. 1996; Audenaert et al. 2002). Likewise, a study by Michavila et al. (2017) reported that a bioactive compound extracted from a *Pseudomonas protegens* CS1 isolate obtained from lemon leaves and identified as enantio-pyochelin was highly active against *Xanthomonas campestris* both in vitro and in planta. The significance of aerugine, a member of an extensive family of benzyl thiazole and thiazoline natural metabolites that also includes pyochelin (Lin et al. 2010; Kaplan et al. 2021), was also relevant. The latter considering the findings of previous studies that reported that aerugine-based treatments based on isolates obtained from culture filtrates of either *Pseudomonas fluorescens* or *Streptomyces fradiae* successfully protected crop plants such as pepper and cucumber against infection by *Phytophthora capsici* and *Colletotrichum orbiculare*, respectively (Lee et al. 2003) and strongly inhibited conidial/zoospore germination of *C. gloeosporioides* and *Phytophthora parasitica* (Apichaisataienchote et al. 2006). A more recent report further identified aerugine as the major active compound responsible for the strong inhibitory activity against *Ralstonia solanacearum* produced by a particular *Streptomyces* strain isolated from the rhizosphere of traditional Chinese medicinal plants (Zeng et al. 2025). Moreover, the results of a related study designed to test the bioactive effect of a strain of *Burkholderia seminalis* against *Fusarium oxysporum* suggested that pyochelin could be acting synergistically with other metabolites, e.g., aerugine and/or other antimicrobial byproducts of pyochelin and enantio-pyochelin biosynthesis, that were also naturally found in the culture medium (da Silva Araújo et al. 2017). Subsequent studies also detected this metabolite in other species of *Burkholderia* (Depoorter et al. 2021; Jaiyesimi et al. 2021), in addition to the phyochelin methyl ester herewith reported (Jaiyesimi et al. 2021). However, the presence of this metabolite was deemed not to have originated naturally, leading to the suggestion that the signal detected could be otherwise representative of thiazostatin A/B, an antioxidant compound which is also a member of the benzyl thiazole and thiazoline family of bioactive natural products, mentioned above, that are characteristically synthesized by *Pseudomonas* and *Burkholderia* bacteria (Lin et al. 2010; Kaplan et al. 2021).

Additionally, ornibactin was reported to be strictly required for the full expression of the bactericidal activity exerted by *B. contaminans* MS14 against several phytopathogens, including *Erwinia amylovora* and *Xanthomonas citri*, thereby suggesting that this siderophore might have an alternative biological function in addition to iron sequestration (Deng et al. 2017). However, and contrary to the proposal of the present study, ornibactin mutants did not affect the antifungal activity of *B. contaminans* MS14 against *Geotrichum candidum*, which was attributed, instead, to the occidiofungin glycopeptide (Wang et al. 2016; Deng et al. 2017). Likewise, ornibactin derivatives synthesized by *B. catarinensis* 89 T had no antifungal activity (Bach et al. 2017), while the deletion of the *cysB* gene in *Burkholderia pyrrocinia* JK-SH007 negatively affected the synthesis of cysteine and of the ornibactin siderophore as well as its antifungal activity (Yu et al. 2023). However, the loss of antifungal activity recorded in the latter study could not be positively linked to the lack of ornibactin C6 and C8 and was otherwise attributed to metabolites produced via iron-sulfur metabolic pathways, which may have also contributed to the reduced anthracnose symptoms observed in the CFSs-treated post-harvest avocados described in the present study. Therefore, additional experimentation using purified siderophore preparations is an aspect that needs to be addressed to confirm the efficacy of the antifungal effect exerted by *Burkholderia* siderophores.

The evidence herewith presented is congruent with the concept of the use of *Burkholderia* strains as a promising alternative for the bio-control of agronomically important phytopathogenic fungi. However, several critical aspects must be considered before their efficient and large-scale use in the field may be implemented (Yusuf et al. 2026). It should be acknowledged that temperature, pH, and nutrient concentration are conditions that may compromise the production of antifungal metabolites and the biocontrol efficacy of *Burkholderia* strains. There is also the need to explore the effects caused by their exposure to varying environmental conditions, which may alter the synthesis of bioactive compounds, antifungal activity, and/or root colonization capacity. An additional aspect requiring further consideration is the persistence of *Burkholderia* strains in the environment, which may have potential long-term effects on soil ecosystems and microbial communities and/or promote competition for ecological niches with other established biocontrol agents such as *Trichoderma* and *Bacillus*. Another point to ponder is the potential health risks associated with the use of these microorganisms, such as the opportunistic pathogenicity identified in certain strains, e.g., *B*. *cenocepacia*, and their prevalent antibiotic resistance mechanisms that may be mobilized to other taxa via horizontal gene transfer. Several actions have been suggested to improve these limiting scenarios. They include genomic characterization to exclude virulence and mobilizable antibiotic resistance loci coupled to rigorous in vitro and in vivo safety testing. Furthermore, mining of *Burkholderia* genomes is predicted to lead to the discovery of novel biocontrol compounds, while genetic manipulation remains an option to improve the antifungal activity, stress tolerance, and plant colonization abilities of these microorganisms and to allow the heterologous expression of biosynthetic gene clusters to non-pathogenic hosts. Finally, a close collaboration among industry, academia, and legislators must be prioritized to facilitate not only the development and commercialization of biocontrol technologies but to permit the successful resolution of regulatory complexities that obstruct their widespread application.

## Conclusions

This study’s findings support the increasing importance of the genus *Burkholderia* as a bio-control agent. They showed that CFSs produced by *B. contaminans* MSR2 represent an effective alternative for the post-harvest protection of avocado fruits against the highly damaging anthracnose disease, which, nowadays, strongly relies on the use of the synthetic prochloraz fungicide. Congruent with several other previous reports, the metabolomic data of this study support the role of pyochelin, and possibly ornibactin, siderophores, in addition to aerugine, as major contributing factors for the suppression of anthracnose disease observed in avocado fruits treated with *B. contaminans* MSR2 siderophore-enriched CFEs. However, the antifungal effect contribution of several other chemically diverse metabolites secreted by *B. contaminans* MSR2 under iron-limited conditions, whose presence was suggested by their untargeted metabolomic analysis, cannot be ruled out.

## Supplementary information

Below is the link to the electronic supplementary material.

ESM 1 (PDF 1.14 MB)

## Data Availability

The datasets generated during and/or analyzed during the current study are available from the corresponding author on reasonable request. LC–MS/MS raw data (mzML) are available in the public database MassIVE under the ID (V2 6f7b9469).
