# Supplementary material for: Burkholderia contaminans siderophore-rich supernatants suppress post-harvest anthracnose in avocado fruits
Source: Appl Microbiol Biotechnol. 2026 Jan 23;110:35. doi: 10.1007/s00253-026-13715-2 (PMC12831694; doi:10.1007/s00253-026-13715-2)
Supplement: Supplementary file 1 [file 253_2026_13715_MOESM1_ESM.pdf]

Supporting information

Applied Microbiology and Biotechnology

Burkholderia contaminans´ siderophore-rich supernatants suppress post-harvest anthracnose in avocado fruits

Coyolxauhqui Barrera-Galicia[1], Sergio A. Covarrubias[1], Juan Armando Flores de la Torre[1], Héctor A. Peniche-Pávia[3], John P. Délano-Frier[2*]

[1]Universidad Autónoma de Zacatecas, Facultad de Ciencias Químicas. Carretera Zacatecas-Guadalajara Km. 6, ejido "La Escondida", C.P. 98160. Ciudad Universitaria campus Siglo XXI, Zacatecas, Zacatecas.

[2]Departamento de Biotecnología y Bioquímica, Centro de Investigación y de Estudios Avanzados del IPN. Km 9.6 Libramiento Norte Carretera Irapuato-León, C.P. 36824. Irapuato, Guanajuato, México.

[3]Departamento de Recursos del Mar, Cinvestav-IPN, Km. 6 antigua carretera a Progreso Apdo. Postal 73, Cordemex, 97310, Mérida, Yucatán., México.

* Corresponding author
John Délano-Frier
john.delano@cinvestav.mx
Tel.: +52-462-623-9636
ORCID-ID: 0000-0003-4010-4464

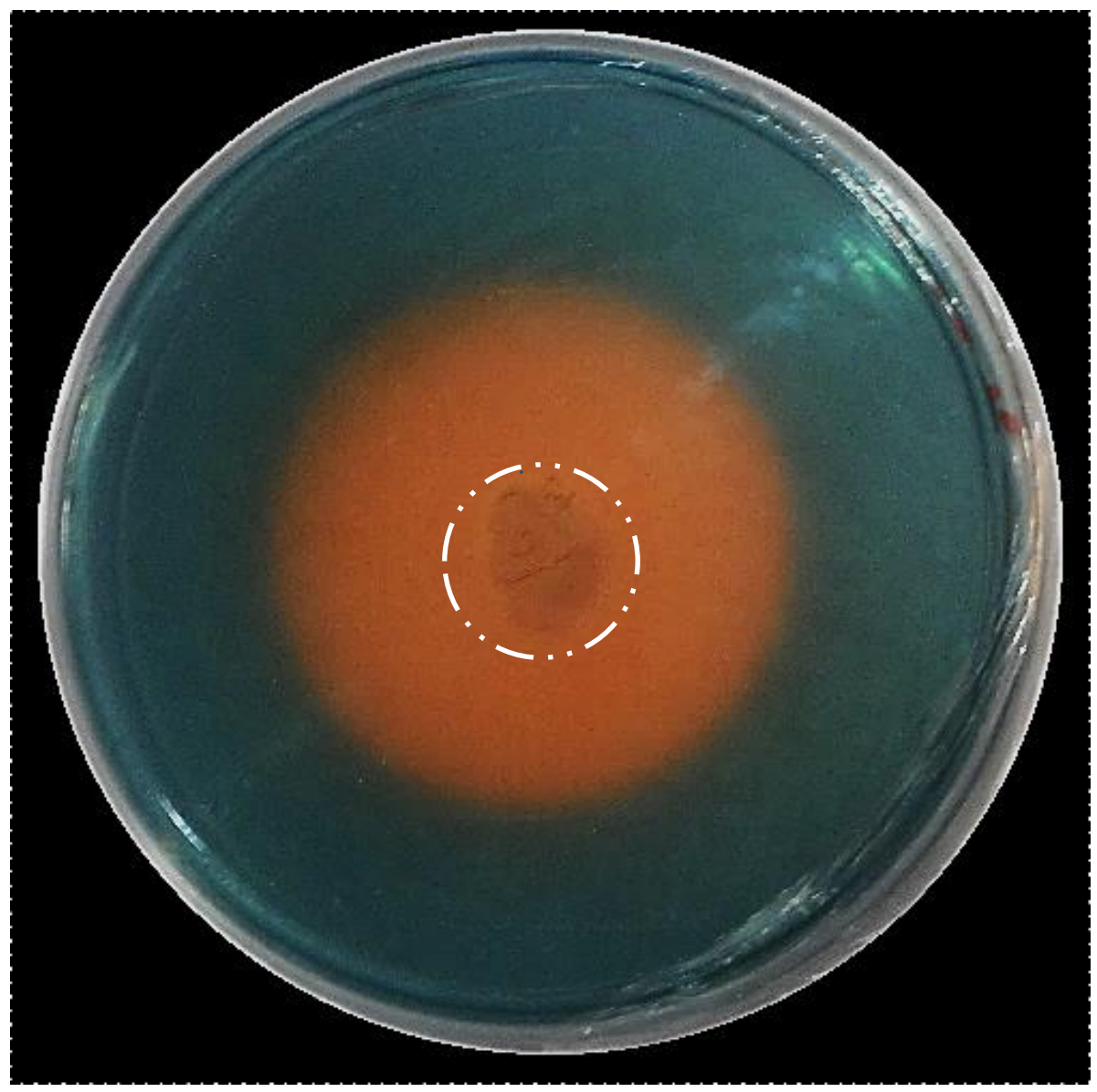

Fig. S1 The formation of a characteristic orange halo in CAS agar media was indicative of siderophore production by the *B. contaminans* MSR2 strain (colony boundary is indicated with a circle) used to test its antagonism toward *C. gloeosporioides* growth

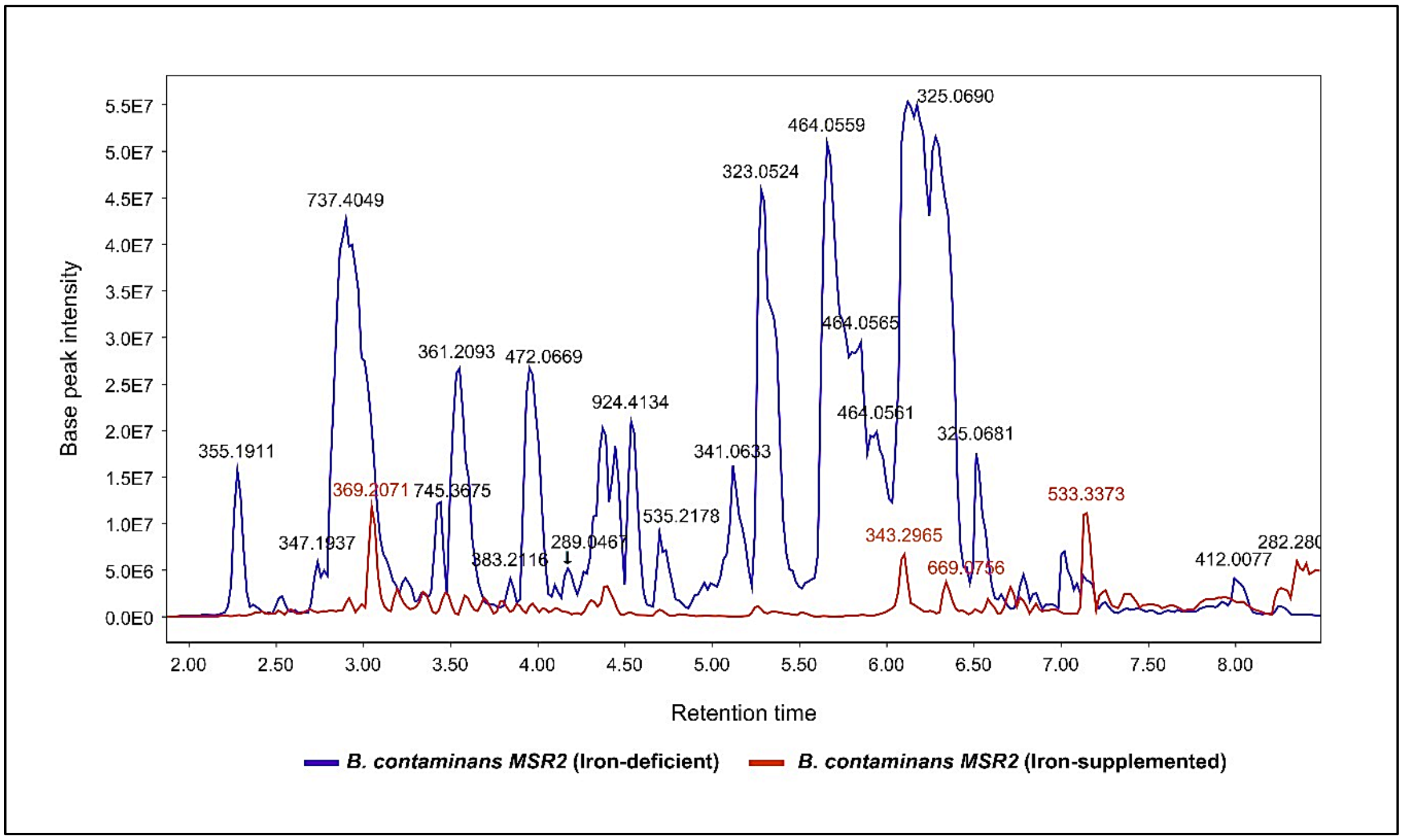

Fig. S2 Total ion chromatograms (TICs) showing the base peak intensity of extracellular metabolites produced by *B. contaminans* MSR2 under iron-deficient (blue line) and iron-supplemented (red line) conditions. Chromatograms were obtained by LC-MS in positive ionization mode

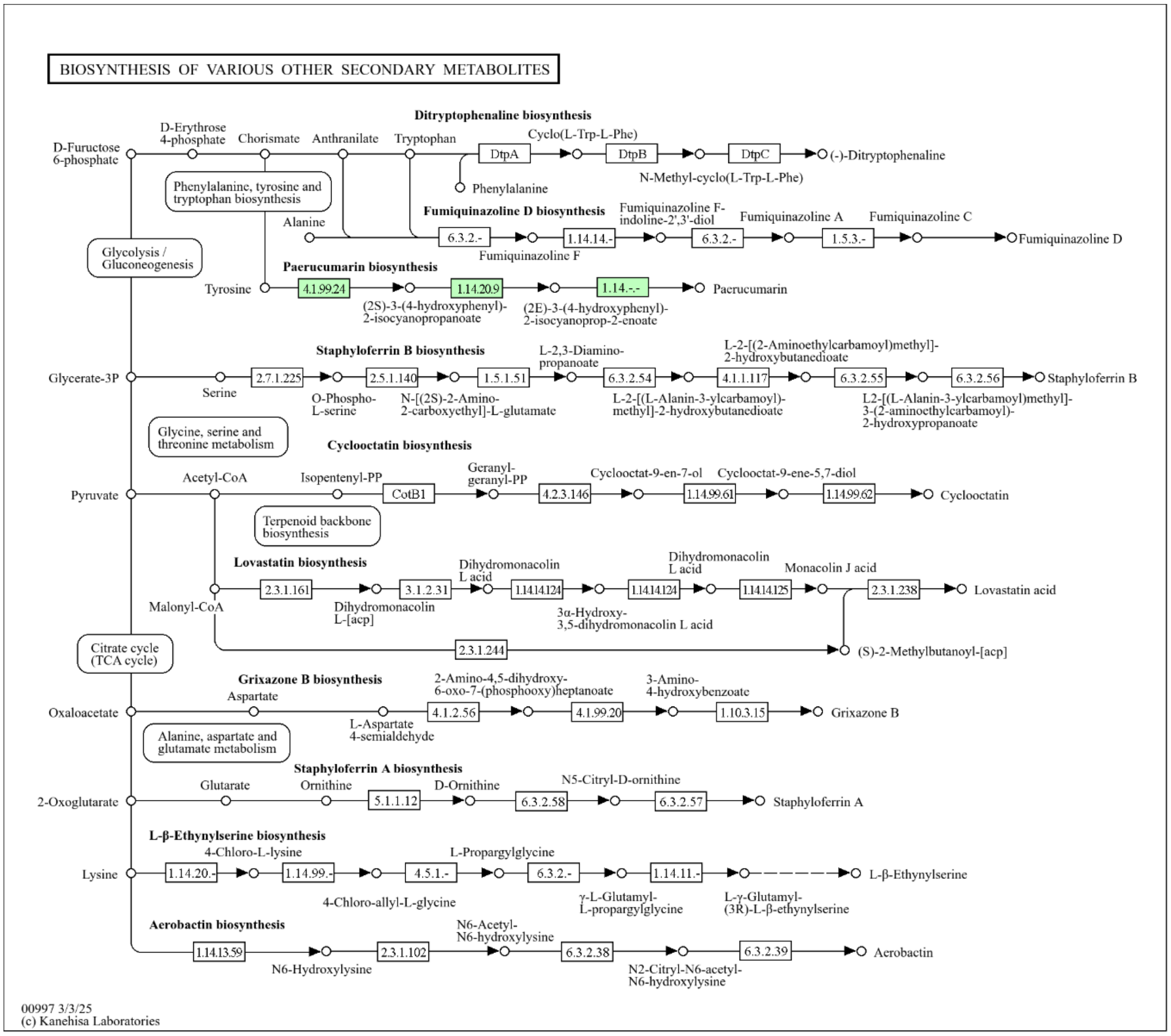

Figure S3 KEGG Diverse secondary metabolites biosynthetic pathway from *B. mallei* ATCC 23344 (entry: bma00997). The map was adapted from the KEGG database (accessed July 2025) to contextualize the experimentally detected compounds.

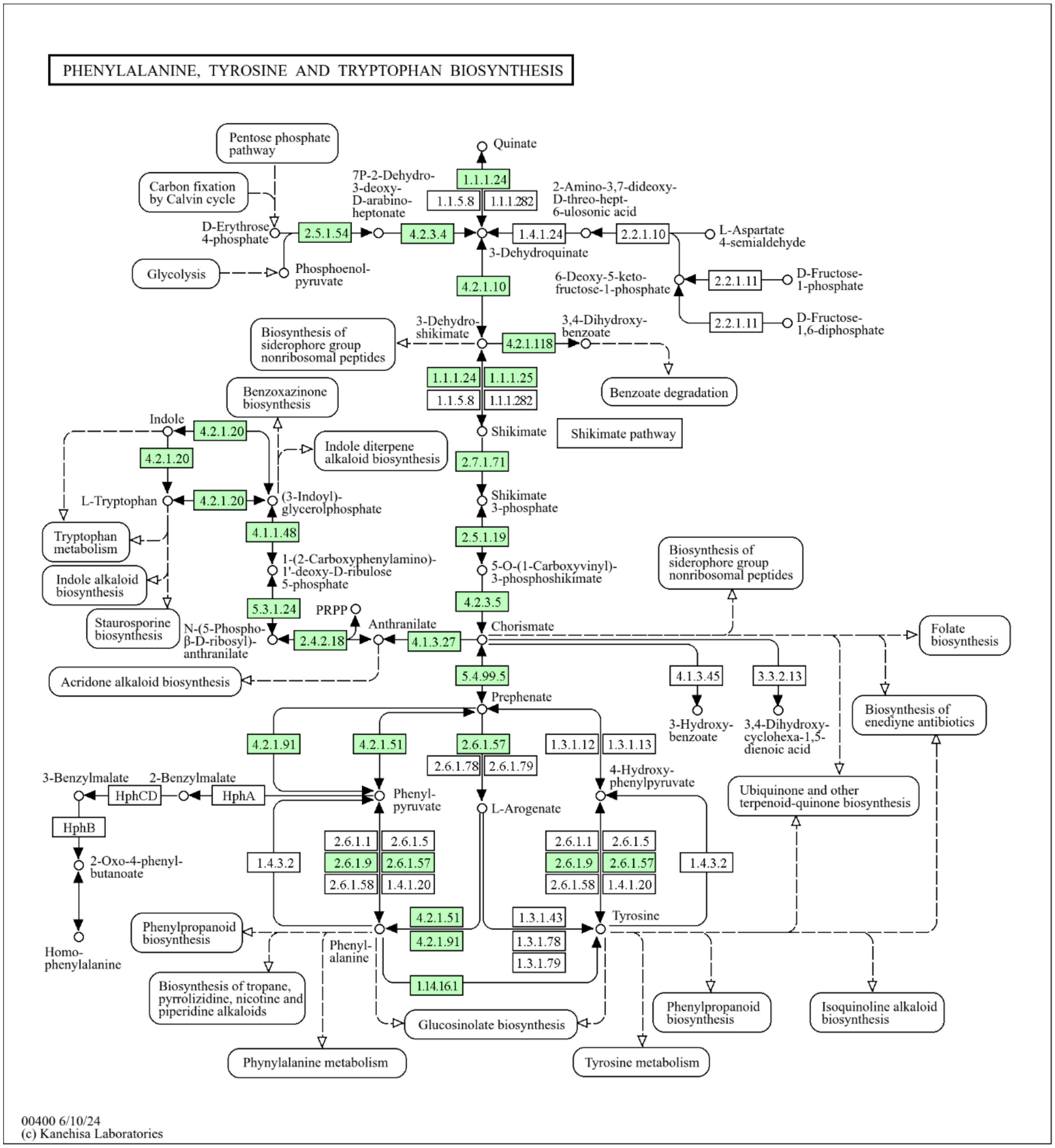

Figure S4. KEGG pathway map of phenylalanine, tyrosine and tryptophan biosynthesis from *B. mallei* ATCC 23344 (entry: bma00400). The map was adapted from the KEGG database (accessed July 2025) to contextualize the experimentally detected compounds.

Table S1. Secondary metabolites known to be synthesized by *Burkholderia sensu stricto* bacteria

No.	Name	Formula	Mass	[M+H]	[M+Na]	Reference
1	(*E*)-2-(Hept-2-en-1-yl) quinolin-4(1H)-one	$C_{16}H_{19}NO$	241.14666	242.15449	264.13643	Li et al. 2018 (doi.org/10.3762/bjoc.14.122)
2	(*E*)-2-(Non-2-en-1-yl) quinolin-4(1H)-one	$C_{18}H_{24}NO$	270.18579	271.19361	293.17556	Li et al. 2018 (doi.org/10.3762/bjoc.14.122)
3	2-Hydroxymethyl-chroman-4-one	$C_{10}H_{10}O_3$	178.06300	179.07082	201.05277	Khan et al. 2004 (doi.org/10.7164/antibiotics.57.726)
4	2-Imino-3-methylene-5-(carboxy-L-threoninyl)-pyrrolidine	$C_{10}H_{15}N_3O_4$	241.10626	242.11408	264.09603	Mitchell et al., 2005 (doi:10.1039/b509319h)
5	2-Imino-3-methylene-5-(carboxy-L-valyl)-pyrrolidine	$C_{11}H_{17}N_3O_3$	239.12699	240.13482	262.11676	Mitchell et al. 2005 (doi:10.1039/b509319h)
6	3-[L-Alanyl-L-homoserinyl-L-aspartyl-β-carboxy]-4-hydroxy-5-oxopyrazole	$C_{14}H_{21}N_5O_9$	403.13393	404.14175	426.12370	Mitchell et al. 2008 (doi:10.1016/j.phytochem.2008.08.013)
7	Aerugine	$C_{10}H_{11}NO_2S$	209.05105	210.05888	232.04082	Rodríguez-Cisneros et al., 2023 (doi.org/10.3390/molecules28041646)
8	Aeruginaldehyde	$C_{10}H_7NO_2S$	205.01975	206.02758	228.00952	PubChem CID 136259032
9	Arsinothricin	$C_5H_{12}O_4NAs$	224.99823	226.00605	247.98800	doi.org/10.1071/EN14247
10	Bactobolin	$C_{14}H_{20}Cl_2N_2O_6$	382.06984	383.07767	405.05961	Greenberg et al. 2020 (doi: 10.1021/acs.jnatprod.9b01237)
11	Bolagladin A	$C_{39}H_{63}N_5O_{14}$	825.43716	826.44498	848.42693	Dashti et al., 2020 (doi.org/10.1002/ange.202009110)
12	Bolagladin B	$C_{40}H_{65}N_5O_{14}$	839.45280	840.46063	862.44257	Dashti et al. 2020 (doi.org/10.1002/ange.202009110)
13	Bongkrekic acid	$C_{28}H_{38}O_7$	486.26176	487.26958	509.25153	PubChem CID: 6433556
14	Bulgecin A	$C_{16}H_{29}N_3O_{14}S_2$	551.10910	552.11693	574.09887	Tomoshige et al. 2018 (doi: 10.1021/acsinfecdis.8b00105)
15	Bulgecin B	$C_{17}H_{27}N_3O_{13}S$	513.12646	514.13429	536.11623	Tomoshige et al. 2018 (doi: 10.1021/acsinfecdis.8b00105)
16	Bulgecin C	$C_{14}H_{24}N_2O_{12}S$	444.10500	445.11283	467.09477	Tomoshige et al. 2018 (doi: 10.1021/acsinfecdis.8b00105)
17	Burkholdine 1215	$C_{52}H_{85}N_{11}O_{22}$	1215.58707	1216.59489	1238.57684	Lin et al. 2012 (doi/10.1021/np300108u)

Table S1 (continued). Secondary metabolites known to be synthesized by *Burkholderia sensu stricto* bacteria

No.	Name	Formula	Mass	[M+H]	[M+Na]	Reference
18	Burkholdine 1119	$C_{52}H_{85}N_{11}O_{21}$	1199.59215	1200.59998	1222.58192	Lin et al. 2012 (doi/10.1021/np300108u)
19	Burkholdine 1213	$C_{52}H_{83}N_{11}O_{22}$	1213.57142	1214.57924	1236.56119	Lin et al. 2012 (doi/10.1021/np300108u)
20	Burkholone	$C_{18}H_{23}NO$	269.17796	270.18579	292.16773	Mori et al. 2007 (doi: 10.1038/ja.2007.92)
21	Burriogladin A	$C_{49}H_{68}N_8O_{12}$	960.49567	961.50350	983.48544	Dose et al. 2021 (doi.org/10.1002/cbic.202100330)
22	Caryoynencin	$C_{18}H_{16}O_3$	280.10995	281.11777	303.09972	Dose et al, 2021 (doi.org/10.1002/cbic.202100330)
23	Cepabactin	$C_7H_9NO_3$	155.05824	156.06607	178.04801	Jiro et al. 1979 (ChemSpider ID: 152678)
24	Cepaciachelin	$C_{24}H_{32}N_4O_7$	488.22710	489.23493	511.21687	Barelmann et al. 1996 (doi.org/10.1515/znc-1996-9-1004)
25	Cepaciamide A	$C_{40}H_{74}N_2O_5$	662.55977	663.56760	685.54954	Jiao et al. 1996 (doi.org/10.1016/0040-4039(95)02342-9), Toshima et al. 1999 (doi.org/10.1016/S0040-4020(99)00248-3); PubChem CID 10723054
26	Cepaciamide B	$C_{21}H_{40}O_3N_2$	368.30389	369.31172	391.29366	Jiao et al. 1996 (doi.org/10.1016/0040-4039(95)02342-9)
27	Cepacin A	$C_{16}H_{14}O_4$	270.08921	271.09704	293.07898	PubChem CID 6435506
28	Cepacin B	$C_{16}H_{14}O_5$	286.08413	287.09195	309.07390	Parker et al. 1984; (DOI: 10.7164/antibiotics.37.431)
29	Cepafungin	$C_{28}H_{46}N_4O_6$	534.34174	535.34956	557.33151	PubChem CID 6439302
30	Enacyloxin IIa	$C_{33}H_{45}Cl_2NO_{11}$	701.23697	702.24479	724.22674	PubChem CID: 5288163
31	Enacyloxin IIa	$C_{33}H_{45}NO_{11}Cl_2$	701.23697	702.24480	724.22674	Dose et al. 2021 (doi.org/10.1002/cbic.202100330)
32	Endolide A	$C_{27}H_{38}N_4O_6$	514.27914	515.28696	537.26891	Almeida et al. 2016 (doi: 10.1021/acs.orglett.5b03553)
33	Endolide B	$C_{28}H_{34}N_4O_7$	538.24275	539.25058	561.23252	Almeida et al. 2016 (doi: 10.1021/acs.orglett.5b03553)
34	FK228	$C_{24}H_{36}N_4O_6S_2$	540.20763	541.21546	563.19740	Liu et al. 2012 (doi.org/10.1107/S160053681203601X)
35	FR900359	$C_{49}H_{75}N_7O_{15}$	1001.53212	1002.53994	1024.52189	Fujioka et al. 1988 (doi/10.1021/jo00247a030)
36	FR901463	$C_{27}H_{42}ClNO_8$	543.25990	544.26772	566.24967	Nakajima et al. 1996 (doi: 10.7164/antibiotics.49.1196)

Table S1 (continued). Secondary metabolites known to be synthesized by *Burkholderia sensu stricto* bacteria

No.	Name	Formula	Mass	[M+H]	[M+Na]	Reference
37	FR901464	$C_{27}H_{41}NO_8$	507.28322	508.29104	530.27299	Nakajima et al. 1996 (doi: 10.7164/antibiotics.49.1196)
38	FR901465	$C_{27}H_{41}NO_9$	523.27813	524.28596	546.26790	Nakajima et al. 1996 (doi: 10.7164/antibiotics.49.1196)
39	Gladiofungin A	$C_{27}H_{39}NO_8$	505.26757	506.27539	528.25734	Dose et al. 2021 (doi.org/10.1002/cbic.202100330)
40	Gladiolin	$C_{44}H_{74}O_{11}$	778.52312	779.53094	801.51289	Song et al. 2017 (doi: 10.1021/jacs.7b03382)
41	Glidobactin A	$C_{27}H_{44}N_4O_6$	520.32609	521.33391	543.31586	Oka et al. 1988 (doi.org/10.7164/antibiotics.41.1338)
42	Glidobactin B	$C_{29}H_{46}N_4O_6$	546.34174	547.34956	569.33151	Oka et al. 1988 (doi.org/10.7164/antibiotics.41.1338)
43	Glidobactin C	$C_{29}H_{48}N_4O_6$	548.35739	549.36521	571.34716	Oka et al. 1988 (doi.org/10.7164/antibiotics.41.1338)
44	Glidobactin D	$C_{27}H_{44}N_4O_7$	536.32100	537.32883	559.31077	PubChem CID: 6444158
45	Glidobactin F	$C_{25}H_{40}N_4O_6$	492.29479	493.30261	515.28456	PubChem CID: 6444157
46	Glidobactin H	$C_{20}H_{30}N_4O_6$	422.21654	423.22436	445.20631	Oka et al. 1988 (doi.org/10.7164/antibiotics.41.1338)
47	Glidopeptin A	$C_{60}H_{102}N_{16}O_{19}$	1350.75072	1351.75854	1373.74049	Dose et al. 2021 (doi.org/10.1002/cbic.202100330)
48	Gramibactin	$C_{32}H_{55}O_{16}N_{10}$	835.37976	836.38758	858.36953	Hermenau et al. 2018 (doi.org/10.1038/s41589-018-0101-9)
49	Haereogladin A	$C_{44}H_{54}N_6O_{12}$	858.37997	859.38780	881.36974	Dose et al. 2021 (doi.org/10.1002/cbic.202100330)
50	Heptarhizin	$C_{35}H_{54}O_{10}N_7$	732.39322	733.40104	755.38299	Niehs et al. 2018 (doi.org/10.1002/cbic.201800400)
51	Holrhizin A	$C_{42}H_{68}N_6O_{10}$	816.49969	817.50752	839.48946	PubChem CID 146684039
52	Icosalide A	$C_{36}H_{64}N_4O_{10}$	712.46225	713.47007	735.45202	Dose et al. 2021 (doi.org/10.1002/cbic.202100330)
53	Iodinin	$C_{12}H_8N_2O_4$	244.04841	245.05623	267.03818	Compound CID: 135441735
54	Kirkamide	$C_9H_{15}NO_5$	217.09502	218.10285	240.08479	Sieber et al. 2015 (dx.doi.org/10.1002/anie.201502696.)

Table S1 (continued). Secondary metabolites known to be synthesized by *Burkholderia sensu stricto* bacteria

No.	Name	Formula	Mass	[M+H]	[M+Na]	Reference
55	Lagriamide	$C_{41}H_{68}N_2O_{10}$	748.48740	749.49522	771.47717	Compound CID: 146684558
56	Lagriene	$C_{44}H_{74}O_{11}$	778.52312	779.53094	801.51289	Flórez et al. (doi: 10.1038/ncomms15172)
57	L-Alanyl-L-homoserinyl-L-aspartic acid	$C_{11}H_{19}N_3O_7$	305.12230	306.13013	328.11207	Mitchell et al. 2008 (doi:10.1016/j.phytochem.2008.08.013)
58	Maculosin	$C_{14}H_{16}N_2O_3$	260.11609	261.12392	283.10586	Yap et al. 2016
59	Malleilactone	$C_{18}H_{26}O_4$	306.18311	307.19094	329.17288	Biggins 2013 (doi: 10.1021/ja3052156) PubChem CID: 136229184
60	Malleobactin E	$C_{24}H_{42}N_8O_{13}$	650.28714	651.29496	673.27691	Dashti et al. 2020 (doi.org/10.1002/ange.202009110)
61	Malleonitrone	$C_{33}H_{47}N_9O_{13}S$	809.30141	810.30923	832.29118	Trottmann et al. 2019 (doi.org/10.1002/anie.201811131.)
62	Methyl myristate	$C_{15}H_{30}O_2$	242.22458	243.23241	265.21435	Yap et al. 2016
63	Occidiofingin A	$C_{54}H_{79}N_{12}O_{19}$	199.55845	200.56627	222.54822	Lu et al. 2010 (doi: 10.1021/bi900814c)
64	Occidiofingin B	$C_{54}H_{79}N_{12}O_{20}$	1215.55336	1216.56119	1238.54313	Lu et al. 2010 (doi: 10.1021/bi900814c)
65	Onychocin D	$C_{31}H_{42}N_4O_4$	534.32061	535.32843	557.31038	Almeida et al. 2018 (doi.org/10.1128/AEM.00660-18)
66	Ornibactin C4	$C_{26}H_{48}N_8O_{13}$	680.33409	681.34191	703.32386	PubChem CID 133053363; Stephan et al. 1993 (10.1007/BF00140109)
67	Ornibactin C6	$C_{28}H_{52}N_8O_{13}$	708.36539	709.37321	731.35516	PubChem CID 133053447; Stephan et al. 1993 (10.1007/BF00140109)
68	Ornibactin C8	$C_{30}H_{56}N_8O_{13}$	736.39669	737.40451	759.38646	Stephan et al. 1993 (10.1007/BF00140109)
69	Pityriacitrin	$C_{20}H_{13}N_3O$	311.10586	312.11369	334.09563	Mayser et al. 2002 (doi 10.1007/s00403-002-0294-2)
70	Pyochelin	$C_{14}H_{16}N_2O_3S_2$	324.06024	325.06806	347.05001	PubChem CID 443588
71	Pyochelin-methylester	$C_{15}H_{19}N_2O_3S_2$	339.08371	340.09154	362.07348	Lybbert et al. 2020 (oi.org/10.3390/metabo10110445)
72	Pyrrolnitrin	$C_{10}H_6C_{l2}N_2O_2$	255.98063	256.98846	278.97040	PubChem CID 13916
73	Rhizobitoxine	$C_7H_{14}N_2O_4$	190.09536	191.10318	213.08513	PubChem CID 6436603
74	Rhizomide A	$C_{35}H_{53}N_7O_{10}$	731.38539	732.39322	754.37516	Lepetit et al. 2023 (doi.org/10.1016/j.bmcl.2023.129506)

Table S1 (continued). Secondary metabolites known to be synthesized by *Burkholderia sensu stricto* bacteria

No.	Name	Formula	Mass	[M+H]	[M+Na]	Reference
75	Rhizonin	$C_{42}H_{65}N_7O_9$	811.48438	812.49220	834.47415	Partida-Martínez et al. 2006 (doi.org/10.1128/AEM.01784-06)
76	Salicylic acid	$C_7H_6O_3$	138.03170	139.03952	161.02147	Compound CID: 338
77	Sinapigladioside	$C_{21}H_{27}NO_9S$	467.12501	468.13283	490.11478	Dose et al. 2021 (doi.org/10.1002/cbic.202100330)
78	Spliceostatin B	$C_{28}H_{41}NO_7$	503.28830	504.29613	526.27807	He et al. 2014 (doi.org/10.1021/np500342m)
79	Spliceostatin C	$C_{28}H_{41}NO_8$	519.28322	520.29104	542.27299	He et al. 2014 (doi.org/10.1021/np500342m)
80	Spliceostatin E	$C_{26}H_{37}NO_6$	459.26209	460.26991	482.25186	He et al. 2014 (doi.org/10.1021/np500342m)
81	Spliceostatin F	$C_{28}H_{42}ClNO_8$	555.25990	556.26772	578.24967	He et al. 2014 (doi.org/10.1021/np500342m)
82	Spliceostatin G	$C_{21}H_{31}NO_6$	393.21514	394.22296	416.20491	He et al. 2014 (doi.org/10.1021/np500342m)
83	Sulfazecin	$C_{12}H_{20}N_4O_9S$	396.09510	397.10293	419.08487	Li et al. 2017 (doi.org/10.1016/j.chembiol.2016.11.010)
84	Thailandamide A	$C_{43}H_{59}NO_8$	717.42407	718.43189	740.41384	Compound CID: 163285733
85	Thailandamide lactone	$C_{43}H_{55}NO_8$	713.39277	714.40059	736.38254	Compound CID: 102047101
86	Thailandepsin A	$C_{23}H_{37}N_3O_6S_3$	547.18445	548.19228	570.17422	Whang et al. 2011; (doi: 10.1021/np200324x); PubChem CID 70698162
87	Thailandepsin B	$C_{24}H_{39}N_3O_6S_2$	529.22803	530.23586	552.21780	Whang et al. 2011; (doi: 10.1021/np200324x); PubChem CID 70698114
88	Thailanstatin A	$C_{28}H_{42}NO_9$	536.28596	537.29378	559.27573	He et al. 2014 (doi.org/10.1021/np500342m)
89	Thailanstatins B	$C_{28}H_{42}ClNO_9$	571.25481	572.26264	594.24458	He et al., 2014 (doi.org/10.1021/np500342m)
90	Toxoflavin	$C_7H_9N_5O_2$	195.07563	196.08345	218.06540	Dose et al. 2021 (doi.org/10.1002/cbic.202100330)
91	Tropolone	$C_7H_6O_2$	122.03678	123.04461	145.02655	PubChem CID 10789

Table S2. Peaks annotated to have MS1 exact mass value that those corresponding to known *B. contaminans* MSR2 secondary metabolites observed in bioactive CFS [Fe0]

m/z	RT (min)	Metabolite	Theorical mass	Adduct	Error ppm
206.02719	6.065	Aeruginaldehyde	206.027576	[M+H]	-1.87
206.02722	4.237	Aeruginaldehyde	206.027576	[M+H]	-1.73
218.10407	2.615	Kirkamide	218.102849	[M+H]	5.60
241.10553	6.776	2-Imino-3-methylene-5-(carboxy-*L*-threoninyl)-pyrrolidine	241.106257	[M]	-3.02
241.10739	6.236	2-Imino-3-methylene-5-(carboxy-*L*-threoninyl)-pyrrolidine	241.106257	[M]	4.70
256.98724	6.777	Pyrrolnitrin	256.988459	[M+H]	-4.74
287.09328	3.804	Cepacin B	287.09195	[M+H]	4.63
325.06795	6.142	Pyochelin	325.068062	[M+H]	-0.34
325.0681	6.53	Pyochelin	325.068062	[M+H]	0.12
325.06856	5.871	Pyochelin	325.068062	[M+H]	1.53
347.04963	6.166	Pyochelin	347.050007	[M+Na]	-1.09
369.31053	6.403	Cepaciamide B	369.311718	[M+H]	-3.22
382.06756	5.224	Bactobolin	382.069844	[M]	-5.98
403.13457	4.006	3-[L-Alanyl-L-homoserinyl-L-aspartyl-b-carboxy]-4-hydroxy-5-oxopyrazole	403.133929	[M]	1.59
423.2234	3.076	Glidobactin H	423.224361	[M+H]	-2.27
444.10285	5.26	Bulgecin C	444.105	[M]	-4.84
460.26912	6.716	Spliceostatin E	460.269914	[M+H]	-1.73
490.11254	3.168	Sinapigladioside	490.114776	[M+Na]	-4.56
521.33241	2.41	Glidobactin A	521.333911	[M+H]	-2.88
523.27562	2.859	FR901465	523.278134	[M]	-4.80
534.34412	4.024	Cepafungin	534.341736	[M]	4.46
535.32444	4.785	Onychocin D	535.328431	[M+H]	-7.46
544.2705	2.759	FR901463	544.267722	[M+H]	5.10
546.27142	4.963	FR901465	546.267904	[M+Na]	6.44

Table S2 (continued). Peaks annotated to have MS1 exact mass value that those corresponding to known *B. contaminans* MSR2 secondary metabolites observed in bioactive CFS [Fe0]

m/z	**RT (min)**	**Metabolite**	**Theorical mass**	**Adduct**	**Error ppm**
548.19734	3.472	Thailandepsin A	548.192278	[M+H]	9.23
548.36007	4.138	Glidobactin C	548.357386	[M]	4.89
557.32846	3.029	Cepafungin	557.331506	[M+Na]	-5.47
563.19562	4.991	FK228	563.1974	[M+Na]	-3.16
574.09513	7.541	Bulgecin A	574.098871	[M+Na]	-6.52
681.33829	1.416	Ornibactin C4	681.341912	[M+H]	-5.32
703.32427	2.859	Ornibactin C4	703.323857	[M+Na]	0.59
703.32492	2.659	Ornibactin C4	703.323857	[M+Na]	1.51
708.36559	3.294	Ornibactin C6	708.365387	[M]	0.29
709.36876	2.681	Ornibactin C4	709.373212	[M+H]	-6.28
709.3729	2.293	Ornibactin C6	709.373212	[M+H]	-0.44
709.3729	2.293	Ornibactin C4	709.373212	[M+H]	-0.44
717.42682	4.504	Thailandamide A	717.424069	[M]	3.83
731.38264	3.74	Rhizomide A	731.385393	[M]	-3.76
732.39206	2.841	Rhizomide A	732.393218	[M+H]	-1.58
732.39206	2.841	Heptarhizin	732.393217	[M]	-1.58
737.40505	2.934	Ornibactin C8	737.404511	[M+H]	0.73
737.40551	3.041	Ornibactin C8	737.404511	[M+H]	1.35
755.37919	3.56	Heptarhizin	755.382987	[M+Na]	-5.03
811.48863	4.163	Rhizonin	811.484378	[M]	5.24
835.38281	7.727	Gramibactin	835.379755	[M]	3.66
839.4528	7.001	Bolagladin B	839.4528	[M]	0.00
858.38143	4.298	Haereogladin A	858.379974	[M]	1.70
859.38484	8.371	Haereogladin A	859.387799	[M+H]	-3.44

Table S3. Significant difference accumulated metabolites in *B. contaminans* MSR2 CFS [Fe0] of the enrichment pathway analysis

	Raw p	Impact	Matched compounds*
O-Antigen nucleotide sugar biosynthesis	1.07E-07	0.76	***N*-Acetyl-alpha-D-glucosamine 1-phosphate**; **CDP-4-dehydro-6-deoxy-D-glucose**; UDP-glucose; **D-glucose 1-phosphate**; **dTDP-4-oxo-6-deoxy-D-glucose**; **dTDP-4-dehydro-beta-L-rhamnose**; **dTDP-glucose**; **D-mannose 6-phosphate**; GDP-4-dehydro-6-deoxy-D-mannose; **GDP-mannose**; **D-mannose 1-phosphate**; **UDP-*N*-acetyl-alpha-D-glucosamine**; **CDP-4-dehydro-3,6-dideoxy-D-glucose**; **UDP-glucuronate**; UDP-alpha-D-galactose; **dTDP-L-rhamnose**; **D-fructose 6-phosphate**; **GDP-6-deoxy-D-talose**
Amino sugar and nucleotide sugar metabolism	4.89E-07	0.92	***N*-Acetyl-D-glucosamine**; ***N*-acetyl-alpha-D-glucosamine 1-phosphate**; UDP-glucose; **D-glucose 1-phosphate**; **UDP-glucuronate**; ***N*-acetyl-D-glucosamine 6-phosphate**; **D-mannose 6-phosphate**; **GDP-mannose**; **D-glucosamine 6-phosphate**; **alpha-D-glucosamine 1-phosphate**; **D-mannose 1-phosphate**; UDP-L-Ara4FN; **UDP-*N*-acetyl-alpha-D-glucosamine**; **UDP-*N*-acetyl-3-(1-carboxyvinyl)-D-glucosamine**; **CDP-4-dehydro-6-deoxy-D-glucose**; **chitobiose**; chitin; GDP-4-dehydro-6-deoxy-D-mannose; D-glucosamine; D-mannose; **alpha-D-glucose 6-phosphate**; D-fructose; alpha-D-glucose; undecaprenyl phosphate alpha-L-Ara4FN; *N*-acetylmuramate; ***N*-acetylmuramic acid alpha-1-phosphate**; ***N*-acetylmuramic acid 6-phosphate**; **D-fructose 6-phosphate**; 1,6-anhydro-*N*-acetyl-beta-muramate; UDP-alpha-D-galactose; **UDP-D-xylose**; **UDP-*N*-acetylmuramate**; **CDP-4-dehydro-3,6-dideoxy-D-glucose**; **GDP-6-deoxy-D-talose**; undecaprenyl phosphate alpha-L-Ara4N
Purine metabolism	2.81E-05	0.71	**GDP**; D-ribose 5-phosphate; L-glutamine; 5-phospho-alpha-D-ribose 1-diphosphate; 5-phosphoribosylamine; **5'-phosphoribosylglycinamide**; 2-(formamido)-*N*1-(5'-phosphoribosyl)acetamidine; **1-(5'-phosphoribosyl)-5-amino-4-imidazolecarboxamide**; 1-(5'-phosphoribosyl)-5-amino-4-(*N*-succinocarboxamide)-imidazole; **1-(5-phospho-D-ribosyl)-5-amino-4-imidazolecarboxylate**; **1-(5'-phosphoribosyl)-5-formamido-4-imidazolecarboxamide**; **ADP**; **dADP**; AMP; ***N*6-(1,2-dicarboxyethyl)-AMP**; **IMP**; adenosine; dAMP; adenine; **IDP**; **xanthosine 5'-phosphate**; hypoxanthine; **GMP**; **inosine**; xanthine; guanine; (*S*)-ureidoglycolate; allantoate; (*S*)-allantoin; urea; guanosine 3',5'-bis(diphosphate); guanosine 3'-diphosphate 5'-triphosphate; dGMP; **dGDP**; **guanosine**; GTP; P1,P4-Bis(5'-adenosyl)tetraphosphate; sulfate; **adenylyl sulfate**; **5'-phosphoribosyl-*N*-formylglycinamide**; ITP; XTP; 3'-AMP; **guanosine 3'-phosphate**; **2',3'-cyclic GMP**; **dGTP**; 2',3'-cyclic AMP; **ADP-ribose**; **dIDP**; carbamoyl phosphate; **aminoimidazole ribotide**; **5-carboxyamino-1-(5-phospho-D-ribosyl)imidazole**; **5-hydroxy-2-oxo-4-ureido-2,5-dihydro-1H-imidazole-5-carboxylate**; alpha-D-ribose 1-phosphate; dITP; **ATP**; ***N*6-succino-2-amino-2'-deoxyadenylate**; dZMP; **dZDP**; dATP; deoxyadenosine; **xanthosine**; glyoxylate; HCO_3; ammonia; (*R*)(-)-allantoin; deoxyguanosine; **3'-phosphoadenylyl sulfate**; urate; **2'-deoxyinosine 5'-phosphate**; **dZTP**
Polyketide sugar unit biosynthesis	0.000159	1	**dTDP-4-dehydro-beta-L-rhamnose**; **dTDP-4-oxo-6-deoxy-D-glucose**; **dTDP-glucose**; **D-glucose 1-phosphate**; **dTDP-6-deoxy-beta-L-talose**; **dTDP-L-rhamnose**
Streptomycin biosynthesis	0.000242	0.97	D-Glucose; **1D-myo-inositol 3-phosphate**; **dTDP-4-dehydro-beta-L-rhamnose**; **dTDP-4-oxo-6-deoxy-D-glucose**; **dTDP-glucose**; **D-glucose 1-phosphate**; myo-inositol; **D-glucose 6-phosphate**; **dTDP-L-rhamnose**; **2,4,6/3,5-pentahydroxycyclohexanone**

*The metabolites highlighted in bold text are those that showed matches with the reference metabolome of *B. mallei* ATCC 23344 from the KEGG library

Table S3 (continued). Significant difference accumulated metabolites in *B. contaminans* MSR2 CFS [Fe0] of the enrichment pathway analysis

	Raw p	Impact	Matched compounds*
Lipopolysaccharide biosynthesis	0.000859	0.50	Lipid IVB; 2,3,2'3'-tetrakis(3-hydroxytetradecanoyl)-D-glucosaminyl-1,6-beta-D-glucosamine 1,4'-bisphosphate; D-arabinose 5-phosphate; **sedoheptulose 7-phosphate**; **D-glycero-alpha-D-manno-heptose 1-phosphate**; D-glycero-alpha-D-manno-heptose 1,7-bisphosphate; **D-glycero-alpha-D-manno-heptose 7-phosphate**; **UDP-*N*-acetyl-alpha-D-glucosamine**; UDP-3-*O*-(3-hydroxytetradecanoyl)-*N*-acetylglucosamine; UDP-3-*O*-(3-hydroxytetradecanoyl)-D-glucosamine; **3-deoxy-D-manno-octulosonate 8-phosphate**; UDP-2,3-bis(3-hydroxytetradecanoyl)glucosamine; **ADP-D-glycero-beta-D-manno-heptose**; **CMP-3-deoxy-D-manno-octulosonate**; **3-deoxy-D-manno-octulosonyl-lipid IV(A)**; **KDO2-lipid IVA**; dodecanoyl-[acyl-carrier protein]; **D-glycero-beta-D-manno-heptose 1-phosphate**; **D-glycero-beta-D-manno-heptose 7-phosphate**; D-glycero-beta-D-manno-heptose 1,7-bisphosphate; **lipid X**; lipid A disaccharide; **3-deoxy-D-manno-octulosonate**; *H. pylori* KDO2-lipid IVA; octadecanoyl-[acyl-carrier protein]; KDO2-lipid A; **deacyl-lipid IVB**; deacyl-lipid IVA; D-ribulose 5-phosphate; **GDP-D-glycero-alpha-D-manno-heptose**; **ADP-L-glycero-beta-D-manno-heptose**; acyl-carrier protein; lauroyl-KDO2-lipid IV(A); stearoyl-KDO2-lipid IVA; **(*S*)-2-hydroxymyristate-modified lipid A**
Histidine metabolism	0.001192	0.78	*N*-Formimino-L-glutamate; *N*-formyl-L-glutamate; 4-imidazolone-5-propanoate; **urocanate**; **L-histidine**; imidazole-4-acetaldehyde; **1-(5-phospho-D-ribosyl)-ATP**; **L-histidinal**; L-histidinol; **L-histidinol phosphate**; **D-erythro-1-(imidazol-4-yl)glycerol 3-phosphate**; **5-(5-phospho-D-ribosylaminoformimino)-1-(5-phosphoribosyl)-imidazole-4-carboxamide**; **phosphoribosyl-AMP**; ***N*-(5'-phospho-D-1'-ribulosylformimino)-5-amino-1-(5''-phospho-D-ribosyl)-4-imidazolecarboxamide**; L-glutamate; imidazole-4-acetate; 5-phospho-alpha-D-ribose 1-diphosphate; **3-(imidazol-4-yl)-2-oxopropyl phosphate**; **1-(5'-phosphoribosyl)-5-amino-4-imidazolecarboxamide**
Acarbose and validamycin biosynthesis	0.012814	1	**D-Glucose 1-phosphate**; **dTDP-glucose**; **dTDP-4-oxo-6-deoxy-D-glucose**
Folate biosynthesis	0.033202	0.44	**Chorismate**; **4-amino-4-deoxychorismate**; THF-polyglutamate; **tetrahydrofolate**; **dihydrofolate**; dihydropteroate; **4-aminobenzoate**; 6-hydroxymethyl-7,8-dihydropterin diphosphate; 6-(hydroxymethyl)-7,8-dihydropterin; 7,8-dihydroneopterin; **7,8-dihydroneopterin 3'-triphosphate**; **2,5-diamino-6-(5'-triphosphoryl-3',4'-trihydroxy-2'-oxopentyl)-amino-4-oxopyrimidine**; **2,5-diaminopyrimidine nucleoside triphosphate**; formamidopyrimidine nucleoside triphosphate; GTP; **folate**; **precursor Z**; 6-carboxy-5,6,7,8-tetrahydropterin; 7-carboxy-7-carbaguanine; 7-cyano-7-carbaguanine; (8*S*)-3',8-cyclo-7,8-dihydroguanosine 5'-triphosphate; **molybdopterin**; adenylated molybdopterin; molybdoenzyme molybdenum cofactor; 4a-hydroxytetrahydrobiopterin; tetrahydrobiopterin; 7,8-dihydrobiopterin; **THF-L-glutamate**; glycolaldehyde; dihydroneopterin phosphate; **6-pyruvoyltetrahydropterin**; 7-aminomethyl-7-carbaguanine; guanylyl molybdenum cofactor; dihydrobiopterin; 7,8-dihydroxanthopterin; 7,8-dihydromonapterin; **2,5-diamino-6-(5-phospho-D-ribosylamino)pyrimidin-4(3H)-one**

*The metabolites highlighted in bold text are those that showed matches with the reference metabolome of *B. mallei* ATCC 23344 from the KEGG library

Table S3 (continued). Significant difference accumulated metabolites in *B. contaminans* MSR2 CFS [Fe0] of the enrichment pathway analysis

	Raw p	Impact	Matched compounds*
Phenylalanine, tyrosine and tryptophan biosynthesis	0.035257	0.72	**5-*O*-(1-Carboxyvinyl)-3-phosphoshikimate**; indoleglycerol phosphate; indole; **1-(2-carboxyphenylamino)-1-deoxy-D-ribulose 5-phosphate**; ***N*-(5-phospho-D-ribosyl)anthranilate**; **chorismate**; **prephenate**; phenylpyruvate; **L-arogenate**; L-phenylalanine; **shikimate 3-phosphate**; shikimate; **3-dehydroquinate**; quinate; **2-dehydro-3-deoxy-D-arabino-heptonate 7-phosphate**; phosphoenolpyruvate; D-erythrose 4-phosphate; L-tyrosine; 3-dehydroshikimate; L-tryptophan; **anthranilate**; 5-phospho-alpha-D-ribose 1-diphosphate; 3-(4-hydroxyphenyl)pyruvate; 3,4-dihydroxybenzoate
Biosynthesis of various other secondary metabolites	0.042311	1	L-Tyrosine; **(2*S*)-3-(4-hydroxyphenyl)-2-isocyanopropanoate**; **(2*E*)-3-(4-hydroxyphenyl)-2-isocyanoprop-2-enoate**; **paerucumarin**
Fructose and mannose metabolism	0.077475	0.49	L-Sorbose; fructan; **mannitol**; D-mannose; D-fructose; **D-mannose 6-phosphate**; GDP-4-dehydro-6-deoxy-D-mannose; **GDP-mannose**; **D-mannose 1-phosphate**; **D-fructose 6-phosphate**; D-glyceraldehyde 3-phosphate; **D-fructose 1-phosphate**; D-fructose 1,6-bisphosphate; L-fuculose 1-phosphate; **D-allose 6-phosphate**; L-fucono-1,5-lactone; 2-dehydro-3-deoxy-L-fuconate; 2,4-diketo-3-deoxy-L-fuconate; beta-L-fucopyranose; **D-sorbitol**; **GDP-6-deoxy-D-talose**; glycerone phosphate; D-glyceraldehyde; (*S*)-lactaldehyde; **D-allulose 6-phosphate**; L-fuconate; (*S*)-lactate
Tryptophan metabolism	0.094706	0.33	**L-Formylkynurenine**; **acetoacetyl-CoA**; crotonoyl-CoA; glutaryl-CoA; 2-oxoadipate; L-kynurenine; formylanthranilate; **3-hydroxy-L-kynurenine**; L-tryptophan; 3-hydroxyanthranilate; indole-3-acetaldehyde; **5-hydroxy-*N*-formylkynurenine**; 5-hydroxyindoleacetaldehyde; (indol-3-yl) acetamide; acetyl-CoA; (*S*)-3-hydroxybutanoyl-CoA; **anthranilate**; **cinnavalininate**; indole-3-acetate; **5-hydroxykynurenine**; **5-hydroxyindoleacetate**
One carbon pool by folate	0.094706	0.94	**Folate**; **dihydrofolate**; **5,10-methenyltetrahydrofolate**; **5,10-methylenetetrahydrofolate**; 10-formyltetrahydrofolate; **tetrahydrofolate**; folinic acid; glycine; **dUMP**; **ATP**; L-methionine; *S*-adenosyl-L-homocysteine; choline; betaine aldehyde; 5-methyltetrahydrofolate; L-serine; **dTMP**; *S*-adenosyl-L-methionine; adenosine; L-homocysteine; betaine

*The metabolites highlighted in bold text are those that showed matches with the reference metabolome of *B. mallei* ATCC 23344 from the KEGG library
